# Drought specifically downregulates mineral nutrition: Plant ionomic content and associated gene expression

**DOI:** 10.1002/pld3.402

**Published:** 2022-08-05

**Authors:** Aurélien D'Oria, Galatéa Courbet, Bastien Billiot, Lun Jing, Sylvain Pluchon, Mustapha Arkoun, Anne Maillard, Christine Paysant‐Le Roux, Jacques Trouverie, Philippe Etienne, Sylvain Diquélou, Alain Ourry

**Affiliations:** ^1^ UMR 950 Ecophysiologie Végétale, Agronomie et Nutritions N, C, S, Normandie Université, UNICAEN INRAE Caen France; ^2^ Laboratoire de Nutrition Végétale, Centre Mondial de l'Innovation Le Groupe Roullier Saint‐Malo France; ^3^ Institut des Sciences des Plantes Paris‐Saclay (IPS2), Université de Paris, CNRS INRAE Orsay France

**Keywords:** ionome, iron, known ionomic genes, manganese, molybdenum, nutrient, transport associated genes, water deficit, zinc

## Abstract

One of the main limiting factors of plant yield is drought, and while the physiological responses to this environmental stress have been broadly described, research addressing its impact on mineral nutrition is scarce. *Brassica napus* and *Triticum aestivum* were subjected to moderate or severe water deficit, and their responses to drought were assessed by functional ionomic analysis, and derived calculation of the net uptake of 20 nutrients. While the uptake of most mineral nutrients decreased, Fe, Zn, Mn, and Mo uptake were impacted earlier and at a larger scale than most physiological parameters assessed (growth, ABA concentration, gas exchanges and photosynthetic activity). Additionally, in *B. napus* , the patterns of 183 differentially expressed genes in leaves related to the ionome (known ionomic genes, KIGs) or assumed to be involved in transport of a given nutrient were analyzed. This revealed three patterns of gene expression under drought consisting of up (transport of Cl and Co), down (transport of N, P, B, Mo, and Ni), or mixed levels (transport of S, Mg, K, Zn, Fe, Cu, or Mn) of regulation. The three patterns of gene regulations are discussed in relation to specific gene functions, changes of leaf ionomic composition and with consideration of the crosstalks that have been established between elements. It is suggested that the observed reduction in Fe uptake occurred via a specific response to drought, leading indirectly to reduced uptake of Zn and Mn, and these may be taken up by common transporters encoded by genes that were downregulated.

## Highlight

In wheat and rapeseed, drought downregulates the uptake of Fe, Mo, Zn, and Mn before reduction of growth, and this was associated with differential expressions of transport‐related genes.

## 1 INTRODUCTION

Availability of water is often described as the major limiting factor in agriculture for plant growth and yield, and global climate change (Seneviratne et al., 2006) is expected to further threaten water resources and rainfall frequency (Dai, 2013; Mishra & Singh, 2010; Wilhite, 2016). The atmospheric fluctuations (i.e., CO_2_, temperature, or rainfall) that cause plant abiotic stress are also reported to disturb the mineral content in plants (Bouchereau et al., 1996; Fan et al., 2008; Fischer et al., 2019; Loladze, 2014; Soares et al., 2019) and thus challenge food safety (Allen, 2000; Rawat et al., 2013) through negative impacts on nutritional quality. Therefore, knowledge of water limitation and its effects on morphological, physiological, biochemical, and molecular traits have been extensively documented in order to guide breeding for suitable characters (Farooq et al., 2009; Nadeem et al., 2019; Raza et al., 2017; Simova‐Stoilova et al., 2016; Tardieu, 2012; Verslues et al., 2006; Zhu et al., 2016). Indeed, water stress, which can be defined as an imbalance between available water in the soil and the plant transpiration demand (Tardieu et al., 2011), causes decreases in the transpiration rate, photosynthesis and ultimately biomass production. The resulting loss in yield is dependent on the species and genotype as well as the intensity and duration of the stress (Bray, 1997; Chaves et al., 2003; Farooq et al., 2009; Levitt, 1980). In response, plants cope with water limitation thanks to processes involving assimilate partitioning, solute accumulation, antioxidant synthesis and hormonal regulation, which can occur in combination (Basu et al., 2016; Farooq et al., 2009; Lee et al., 2009; Nadeem et al., 2019; Park et al., 2021; Tardieu, 2013).

When affected by water limitation, plants need to maintain turgor and stabilize membrane permeability, maintaining water retention by osmotic adjustments (Wang et al., 2013). This could be partly achieved by changes in ion uptake and distribution (Etienne et al., 2018). Nevertheless, ion uptake can be affected by numerous endogenous and exogenous factors. For example, stomatal closure, which is one of the first responses to drought that can be considered as the starting point for transpiration reduction, leads to an alteration in root‐to‐shoot nutrient translocation by reducing the mass flow and thus root uptake capacities (Farooq et al., 2009; Hu et al., 2007). On the other hand, the reduced level of available water in the soil can lead to a decrease in the mobility (Amtmann & Blatt, 2009) and rate of diffusion of individual nutrients from the soil to the root surface (Hu et al., 2007), and this is partly due to its physiochemical properties. Beyond homeostasis, the 20 elements corresponding to the functional ionome (Baxter, 2009; Lahner et al., 2003; Salt, 2004) are essential or beneficial for plant growth and reproduction, and are integrated into important organic compounds or play key roles in plant metabolism. Therefore, it has been suggested that the whole ionomic composition of plant tissues might serve as a tool to reveal plant physiological status (Baxter, 2015; Baxter et al., 2008; Courbet et al., 2021; DOria et al., 2021; Pii et al., 2015; Salt et al., 2008) because the ionome is considered as a social network of elements, controlled by a network of genes responsible for uptake, binding, transport and sequestration (Baxter, 2009).

Actually, the consequences of water stress on the whole ionomic composition have received little attention so far. Few studies have described nutrient uptake or element content fluctuations under water deficit, leading to the conclusion that nutrient uptake mainly declines when water stress intensifies in many plants such as rice (*Oryza sativa*), corn (*Zea mays*), soybean (*Glycine max*), and wheat (*T. aestivum*) (Hu et al., 2007; Tanguilig et al., 1987). However, with data usually provided, it is difficult to assess whether reductions in mineral uptake or more generally in mineral tissue contents were an effect per se of drought or if they were only a consequence of reduced growth. Only a small number of reports have considered the whole ionomic composition and/or quantified root uptake under drought (Acosta‐Gamboa et al., 2017; Fischer et al., 2019; Hu & Schmidhalter, 2005; Sánchez‐Rodríguez et al., 2010). Nevertheless, all of them have described an ionomic shift whose trend is linked to the severity of the stress applied, and as an example, the elemental composition and seed yield of Arabidopsis (Acosta‐Gamboa et al., 2017) changed across the different water regimes tested, with underaccumulation (Fe, Ca, Mg) and overaccumulation (Na) of elements when water limitation increased. Similarly, even though a moderate water stress decreased nutrient uptake in five cultivars of *Solanum lycopersicum* (Sánchez‐Rodríguez et al., 2010), it resulted in a limited fluctuation in the concentration of foliar macro‐ and micro‐ nutrients, whereas in maize the concentration was increased (Fischer et al., 2019). However, a severe water restriction caused a drop in grain nutrient concentration in the latter (Fischer et al., 2019).

Over the last few decades, “‐omic” high‐throughput techniques have been employed to simultaneously identify and quantify gene expression (“transcriptomics”), metabolites (“metabolomics”) or elemental composition (“ionomics”), to decipher metabolic changes in response to biotic or abiotic stresses. These approaches have successfully identified genes controlling the accumulation of one element or a group of elements in different organisms (Eide et al., 2005; Lahner et al., 2003; Sasaki et al., 2016). Recently, Whitt et al. (2020) suggested a curated list of genes that affect the plant ionome and referred to as “Known Ionomic Genes” (KIGs). This list of genes was initially established from *Arabidopsis thaliana* (136 genes identified so far) according to the availability of published works showing that KO or overexpressor mutants were affected for at least one element of the ionome, and has since been extended to other species (ten species revealing 1588 orthologs). Genes that are included may be primarily involved in ion transport and homeostasis and secondarily in metal ion chelation and other types of transport or responses to ions. Nevertheless, this KIG list is limited by the knowledge available, and according to the authors, is over‐represented with transporter genes and genes involved in altering the accumulation of iron and zinc. Contrastingly, genes potentially involved in Mg and Ca and predominantly related to N transport were under‐represented in this KIG list. While this curated list is usable for deciphering how abiotic stresses affect ionomic composition, its enrichment using gene ontology with genes tagged for ion transport could be relevant to provide more complete information.

Thus, in order to decipher the causal relationships resulting from water restriction, the aims of this work were to assess in two contrasting plant species of high agronomic interest (*B. napus* and *T. aestivum*) the effects of moderate or severe drought on (i) growth coupled with physiological and morphological indicators of water stress (imaging analysis, gas exchange, ^13^C discrimination, phytohormone accumulation) and (ii) net root uptake and tissue contents of the 20 elements forming the functional ionome. Furthermore, in *B. napus*, changes in the leaf ionome triggered by drought were examined in the light of the expression pattern of genes from the KIG list previously established by Whitt et al. (2020) and expanded with the “ion transport” annotated genes obtained from the Gene Ontology. The involvement of relevant genes was then interpreted at the light of change of nutrient uptake and ionomic composition triggered by drought and they were discussed according to previously reported effects of mineral nutrient deficiencies.

## 2 MATERIALS AND METHODS

### 2.1 Plant growth conditions and watering regimes

This study was conducted on wheat (*T. aestivum* cv. Bagou) and rapeseed (*B. napus* cv. Trezzor) grown in greenhouse conditions in the high‐throughput plant phenotyping platform of the Centre Mondial de l'Innovation (Saint‐Malo, France) during June and July of 2019. Seeds were germinated in trays filled with a potting soil mixture (NFU 44551, type 992016F1, *Falienor* S.A., Vivy,. France) of sandy loam (40% v/v) and peat moss (60% v/v) supplemented with clay (40 kg m^−3^) and NPK (0.7 kg m^−3^ PG‐MIX 14‐16‐18) (soil solution: pH 5,9 ± 0.2 and Ec (1/1,5) 0,7 mS cm^−1^) (composition given in Data S1) in a growth chamber (16 h day / 8 h night at 20°C and 18°C, respectively, at 80% relative humidity). After the second leaf emergence, one rapeseed or three wheat seedlings were transplanted into 6.5 L pots (20.6 cm diameter) filled with 5000 g of the potting soil cited above and watered at 80% of field capacity (FC) and were placed in a greenhouse with natural light supplemented with high‐pressure sodium lamps (MST SON‐T PIA Plus 400 W, Philips, Netherlands), to ensure at least 250 μmol m^−2^ s^−1^ of PAR at canopy height (16 h day/8 h night at 25°C/20°C).

Pots were then watered daily with tap water to keep 80% FC and fertilized twice with 100 mL of a modified Hoagland solution (nutrient concentrations within demineralized water were kept balanced for an input equivalent to 40 kg N ha^−1^, composition available in Data S2) to ensure the plants' mineral needs until the end of the experiment according to estimations from previous experiments (DOria et al., 2021; Maillard, Etienne, et al., 2016; Sorin et al., 2015).

**FIGURE 1:**
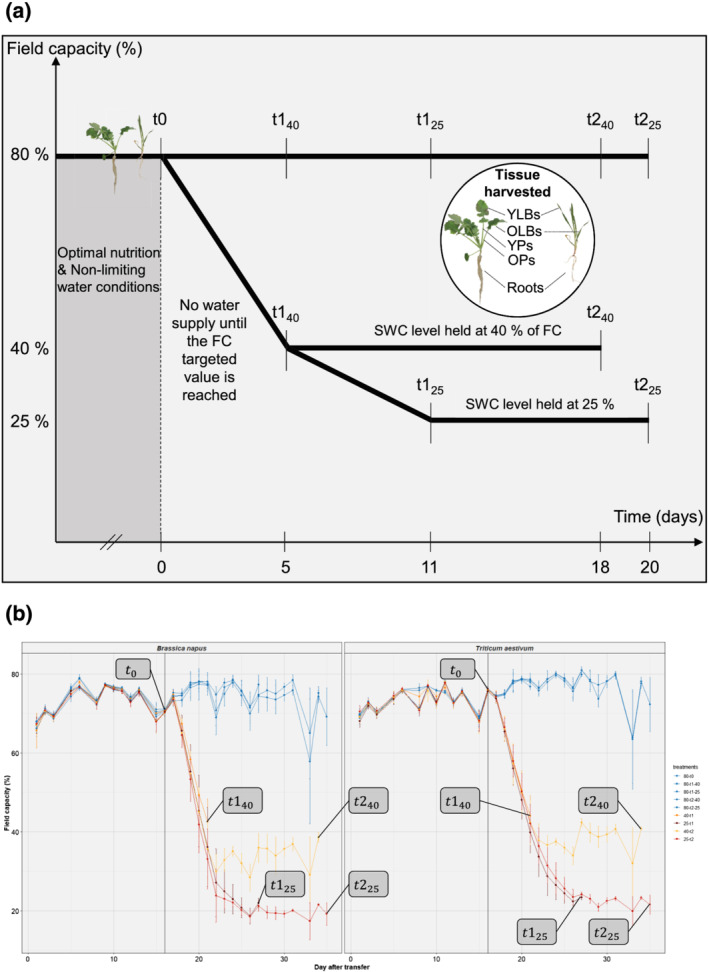
(a) Schematic representation of the experimental design. Thirty days after sowing (t_0_) plants were divided into three subsets: (i) well‐watered control plants were kept at 80% of field capacity (FC) while watering was stopped until the soil water content of all water deficit (WD) pots dropped to (ii) 40% (t1_40_) or (iii) 25% (t1_25_) of FC, which occurred after 5 and 11 days, respectively. These soil water contents were then maintained for 13 days (t2_40_) or 9 days (t2_25_) at 40% and 25% of FC, respectively, using automatic watering. (b) Soil water contents automatically recorded in the high‐throughput phenotyping platform during the experiment. Each value corresponds to the lowest soil water content recorded daily. Control plants (blue) were kept at 80% of FC until the end of the experiment; irrigation of water deficit condition pots was stopped until they reach 40% of FC at t1_40_ (orange) or 25% of FC at t1_25_ (dark red) and then maintained at 40% (yellow) and 25% (red) of FC, respectively, until the final harvests (t2_40_ and t2_25_). Data are given as the mean ± SE (*n* = 10)

Thirty days after sowing (t_0_) plants were divided into three subsets: (*i*) well‐watered control plants that were kept at 80% of FC, and two water deficit (WD) treatments for which watering was stopped until water content (WC) reached (ii) 40% or (iii) 25% of FC, which occurred after 5 (t1_40_) and 11 days (t1_25_), respectively (Figure 1a). At these times (t_0_ and t_1_), one subset of control and corresponding WD pots were harvested. These soil WC levels were then maintained in the remaining pots for 9 days (t2_25_) and 13 days (t2_40_), respectively, so that the t1 and t2 harvest times are considered as representative of short periods and extended periods of WD, respectively. The amount of water to be supplied was determined by automatic weighing twice a day (Figure 1b).

### 2.2 Plant sampling

In order to separate leaves at harvest time that were developed either before water shortage or during water shortage, the last‐developed aboveground tissues (leaves for wheat and petioles for rapeseed) were identified at t_0_ using a marker pen. At each date, five replicates, each consisting of two pots, were harvested. For rapeseed, leaf blades and petioles were separated, and tissues present before water shortage were described as old leaf blades (OLBs) or old petioles (OPs), and newer tissues were designated as young leaf blades (YLBs) and young petioles (YPs). Likewise, for wheat, OLBs and YLBs were considered (Figure 1a). Roots were separated from the shoot and broken roots that remained in the soil were carefully harvested using tweezers. All roots were merged and thoroughly washed with demineralized water. They were only used for mineral content analysis. The number of newly developed leaves from t_0_ and the total leaf number were recorded for rapeseed while the number of tillers was recorded for wheat.

Each fresh sample was weighed and separated into two homogeneous sub‐samples, one immediately frozen in liquid nitrogen and stored at −80°C for molecular analyses and the other was oven dried for 72 h at 65°C for dry weight determination and finally ground for elemental content quantification.

### 2.3 Phenotyping

Plants were monitored using the high‐throughput plant phenotyping platform of the Centre Mondial de l'Innovation (Saint‐Malo, France). An imaging unit acquired images of each plant the day before each harvest. The imaging unit comprises top‐mounted (results not shown in this study) and side‐mounted high‐definition RGB cameras and an LED light system (5500 K ± 500 K). For all pot images, a custom‐made segmentation algorithm based on a machine learning technique was used to determine the mask of the plant and delete the background in order to compute the morphological and color parameters. The morphological parameters measured, which are linked to the development of plant architecture or result from a specific phenotype induced by biotic or abiotic stress, included the convex hull areas and projected areas for the top and side views. For the color parameter, the E × G index was calculated (Excess Green Index Equation 1) as

(1) $$E\times G=\frac{2\times G-\left(R+B\right)}{R+G+B},$$

where *G* (green), *B* (blue), and *R* (red) correspond to mean color intensity by channel.

The net photosynthetic activity of rapeseed and wheat was estimated by instantaneous gas exchange measurements on the last fully‐expanded leaf using a Li‐6800 portable photosynthesis system (LI‐COR, Lincoln, NE, United States) at 500 and 1000 μmol m^2^ s^−1^ of photon flux density (PFD) 1 day before each harvest. Measurement of the CO_2_ assimilation rate (A), transpiration rate (E) and stomatal conductance (g_s_) were performed on the morning after 2 h of illumination with air temperature and humidity in the chamber set to match environmental conditions. The photosynthetic water use efficiency (WUE) was determined as the ratio between A/E.

### 2.4 Elemental content analysis and calculations

Analytical methods used for elemental content quantification were previously detailed in Maillard, Etienne, et al. (2016) and calculation methods in DOria et al. (2021). Briefly, dried samples were ground to a fine powder using 4 mm diameter stainless steel beads in an oscillating grinder (Mixer Mill MM400, Retsch, Haan, Germany). After previous acid (HNO_3_ with H_2_O_2_) digestion with 40 mg of dry weight samples, macronutrients (Mg, P, S, K, and Ca), micronutrients (B, Mn, Fe, Ni, Cu, Zn, and Mo), and beneficial elements (Na, Co, V, and Se) were quantified using high‐resolution inductively coupled plasma mass spectrometry (HR‐ICP‐MS, Element 2™, Thermo Scientific) calibrated with internal and external standards. For total N concentration and ^13^C abundance determination (considered as a long‐term indicator of WUE), 1.5 mg of powder were analyzed by using a continuous flow isotope mass spectrometer (IRMS, Isoprime, GV Instruments, Manchester, United Kingdom) linked to a C/N/S analyzer (EA3000, Euro Vector, Milan, Italy). In order to quantify Cl, Si, and Al, an X‐ray‐fluorescence spectrometer (XEPOS, Ametek, Berwyn, PA, United States) was used with approximately 1 g of dry weight powder using calibration curves obtained from international standards with known concentrations. The quantity (*Q*) of each element in each harvested tissue was calculated using the following equation

(2) $$Q={E_{i}}_{t}\times {{\mathrm{DW}}_{i}}_{t},$$

where *E* is the elemental content (ppm) in a given tissue *i*, at each harvest time *t* (*t*_0_ or *t*′ = t1_40_, t1_25_, t2_40_, or t2_25_) and DW is the corresponding dry weight. The accumulated Net Uptake (NU) from t_0_ of a given element can therefore be estimated by the following equation:

(3) $$\mathrm{NU}=\sum_{i=1}^{n}{Q_{i}}_{t^{\prime }}-\sum_{i=1}^{n}{Q_{i}}_{t_{0}},$$

where *Q* is the amount of an element at *t*_0_ or *t*′ time of the plant tissue *i*, with *n* corresponding to three (roots, OLBs, and YLBs) or five (roots, OLBs, OPs, YLBs, and YPs) tissue types for wheat and rapeseed, respectively. In this study, to assess the specific effect of water deficit (WD) on elemental content or nutrient uptake, some of the results were expressed relative to control, using the ratios: $\frac{E_{\mathrm{WD}}}{E_{\text{control}}}$ or $\;\frac{{\mathrm{NU}}_{\mathrm{WD}}}{NU_{\text{control}.}}$.

### 2.5 Determination of phytohormone profiles

Phytohormone profiles were assessed on rapeseed and wheat leaf samples collected at t1_40_, t1_25_, t2_40_, and t2_25_ and stored at −80°C. Abscisic acid (ABA) and salicylic acid (SA) standards were purchased from Sigma (Lyon, France). The stable isotope‐labeled internal standards, ^2^H_4_‐SA and ^2^H_6_‐ABA, were purchased from OlchemIn (Olomouc, Czech Republic). Twenty milligrams of ground frozen shoots were extracted with 1 mL of 70% methanol (Optima LCMS grade, Fisher, UK) and 1% formic acid (LCMS grade, Fluka analytics, Germany) in Milli‐Q water containing isotope‐labeled internal standards. After extraction, the samples were centrifuged at 12,600 rpm to collect the supernatant. After evaporation (SPE Dry 96, Biotage, Uppsala, Sweden), the extract was resuspended in 2% formic acid solution and purified thanks to an SPE ABN express column of 1 mL/30 mg (Biotage). The phytohormones were eluted with methanol and samples were evaporated and resuspended in a 0.1% formic acid solution before injection into the UPLC‐MS/MS system. The separation and detection were accomplished using a Nexera X2 UHPLC system (Shimadzu, Japan) coupled to a QTrap 6500+ mass spectrometer (Sciex, Canada) equipped with an IonDrive turbo V electrospray source. Phytohormone separation was carried out by injecting 2 μL into a Kinetex Evo C18 core‐shell column (100 × 2.1 mm, 2.6 μm, Phenomenex, USA) at a flow rate of 0.7 mL min^−1^, and the column oven was maintained at 40°C. The mobile phases were composed of solvent A Milli‐Q water containing 0.1% formic acid, and solvent B acetonitrile LCMS grade containing 0.1% formic acid. The separation was achieved with the following gradient: linear gradient from 1 to 60% B over 5 min, 60 to 100% B from 5 to 5.5 min, maintained at 100% B from 5.5 to 7 min, 100 to 1% B from 7 to 7.5 min and maintained at 1% until 9.6 min for column regeneration. The MS analysis was done in scheduled MRM (multiple reaction monitoring) mode in negative mode. The MS acquisition was carried out with the following parameters: ion spray voltage −4500 V; source temperature 600°C; curtain gas 35 psi; nebulizer gas 50 psi; heater gas 60 psi; collision gas medium; entrance potential ‐ 10 V; MRM detection window 30 sec; and target scan time 0.075 sec. The targeted MRM transitions are m/z 136.9/92.9 (SA), 140.9/97.0 (D‐SA), 263.0/153.0 (ABA) and 269.1/159.0 (D‐ABA).

### 2.6 RNA extraction and RNA sequencing (RNA‐seq)

Total RNAs from *B. napus* were extracted from 200 mg FW of leaf blades whose growth occurred after t0 in control plants or during water shortage in WD (YLBs). Samples were ground into powder using liquid nitrogen. The resulting powder was mixed with 750 μL extraction buffer (0.1 M TRIS, 0.1 M LiCl, 1 mM EDTA and SDS 10% [W/V] at pH 8) and 750 mM of hot phenol (80°C, pH 4), then vortexed for 40 s. Chloroform/isoamylalcohol (24:1) was added and the homogenate was centrifuged at 15000 g (5 min at 4°C). The supernatant was thereafter transferred into 750 μL of LiCl solution (4 M) and incubated overnight at 4°C. After centrifugation (15,000 g for 30 min at 4°C), the pellet was suspended in 20 μL of sterile water. Purification of RNAs including a step of DNA digestion by DNAse and was performed using RNA Clean & Concentrator Kits (RCC) according to the manufacturer's protocol (Zymo Research, Irvine, USA).

RNA‐seq libraries were generated with the TruSeq Stranded mRNA protocol (Illumina®, California, U.S.A.) with an average size of 260 bp and were sequenced in paired‐end (PE) mode with a read length of 75 bases on the NextSeq500 with approximately 25 million PE reads per sample. To remove poor quality sequences, classical trimming (Qscore>20, read length>30) was performed and the STAR_2.5.2a mapper was used to align reads against the *B. napus* transcriptome (with local option and other default parameters). The abundance of each of the 101,040 genes (annotation V5 from Genoscope: http://www.genoscope.cns.fr/brassicanapus/data/) was evaluated by unequivocal mapping of the PE reads to each gene. According to this method, 27% of reads were excluded for null counts and 28% of genes with low expression were filtered, which reduced the dataset to around 53,000 genes.

### 2.7 Establishment of the transport‐related and KIGs list

Genes annotated in the “ion transport” gene ontology (GO:0006811, 3018 genes) were extracted from the Amigo2 annotation tool (V 2.5.15) (Carbon et al., 2009) and were complemented with *B. napus* orthologs (448 genes) of the 111 *A. thaliana* KIGs described by Whitt et al. (2020) using the BioMart tool of the EnsemblPlants database (EnsembPlant genes 51—*A. thaliana* genes [Tair10]—*B. napus* genes [AT_PRJEB5043_v1]). The list of 712 *B. napus* genes generated (Data S3) was cleaned and the differentially expressed genes (DEGs) were filtered. Genes from the Whitt et al. (2020) review were assigned with the element(s) defined in the review, while in the case of the GO genes, we only retained those with an unambiguous relationship to the transport of one or more elements in *Arabidopsis* according to the Tair description. Ultimately, 183 genes (of which 103 belonged to the KIG list) were found to be differentially expressed in our experimental conditions considering the two WD treatments compared with the control (Data S3).

### 2.8 Statistical analysis

#### 2.8.1 Univariate analysis

Statistical analyses were performed using R software (version 4.0.3: R Core Team, 2020) and RStudio (version 1.3.1093: RStudio Team, 2020). The variation in assimilation rate, transpiration rate, WUE, stomatal conductance, δ^13^C, side area, convex hull area, excess green index, tissue dry weight, ABA and SA concentrations were analyzed using ANOVA and Tukey's HSD *post‐hoc* test at α = 0.05. Data are given as the mean ± SE (standard error) for *n* = 5. Data were analyzed based on an experimental design that contained five independent replicates, each consisting of a pool of two or six individual plants for rapeseed and wheat, respectively. For each nutrient, net uptake (NU) from t_0_ is given as the mean ± SE for *n* = 25, considering all random subtractive combinations of element quantity between two harvests of five replicates, according to the previously indicated Equation 3.

#### 2.8.2 RNA‐Seq data analysis

Differential analysis of each gene followed the procedure described in Rigaill et al. (2018). Library size was normalized using the trimmed mean of M‐value (TMM) method and the count distribution was modeled with a negative binomial generalized linear model. Dispersion was estimated by the edgeR method (V1.12.0, McCarthy, 2012). Expression differences were tested between WD and control plants using the likelihood ratio test, and p‐values were adjusted by the Benjamini–Hochberg procedure to control False Discovery Rate (FDR, *p*‐value < 0.05). Genes were considered as differentially expressed for an adjusted *p*‐value ≤ 0.05, whatever the absolute value of “Log_2_ fold change”.

Heatmaps were generated using the ComplexHeatmap package (Gu et al., 2016) (version 2.6.2) and multivariate data analysis was performed using the mixOmics package (version 6.14.0).

## 3 RESULTS

### 3.1 Effects of water deficit on plant phenotype

Aboveground and total plant dry weight were not significantly impaired in either *B. napus* or *T. aestivum* plants at 40% of FC after a short (t1_40_) or extended (t2_40_) water deficit, nor after 11 days when the two species faced a drop in WD down to 25% of FC (t1_25_) (Table 1). Overall, plant growth was significantly reduced in both species only after an extended period of severe water restriction (t2_25_). The aboveground biomass (in particular YLBs) and the whole plant biomass of both species were indeed significantly decreased, while root dry weight was significantly reduced only in rapeseed. These biomass reductions were concomitant with a significant decrease in the number of newly‐appeared leaves (YLBs) during WD in rapeseed (decreased by 28% compared with control) as well as with a lower tiller number in wheat (decreased by 29% compared with control) (data not shown). Although differences were not significant, the root biomass of WD plants tended to increase compared with control plants in both species at t2_40_ and t1_25_.

**TABLE 1:** Physiological, and morphological parameters of control plants and plants subjected to short‐term (t1) or extended (t2) (see Materials and Methods for details) water deficit (WD) applied to *Brassica napus* and *Triticum aestivum* plants at 40% or 25% of field capacity (FC)

			*B. napus*				*T. aestivum*				
	Field capacity		40%		25%		40%		25%		
	Harvest time		t1	t2	t1	t2	t1	t2	t1	t2	Unit
Dry weight	YLBs	Control	0.6 ± 0.05	6.5 ± 0.57	3.2 ± 0.3	8.5 ±0.34	0.5 ± 0.05	2.8 ± 0.23	1.4 ± 0.13	3.2 ± 0.19	g.plant^−1^
		WD	0.7 ± 0.06	6.9 ± 0.49	2 ± 0.16**	5.5 ± 0.28**	0.5 ± 0.03	2.2 ± 0.24	1.1 ± 0.11	2 ± 0.08**	
	OLBs	Control	1.7 ± 0.18	3.4 ± 0.12	2.7 ± 0.36	3.3 ± 0.3	0.36 ± 0.03	0.35 ± 0.01	0.35 ± 0.02	0.32 ± 0.02	
		WD	1.7 ± 0.11	2.9 ± 0.16	2.9 ± 0.23	2.7 ± 0.32*	0.33 ± 0.02	0.4 ± 0.02	0.42 ± 0.02	0.35 ± 0.02	
	Aboveground	Control	3.3 ± 0.3	14.9 ± 0.87	8.3 ± 0.98	18.7 ± 0.71	0.9 ± 0.07	3.2 ± 0.24	1.8 ± 0.14	3.5 ± 0.2	
		WD	3.5 ± 0.25	14.5 ± 0.99	7.2 ± 0.5	12.3 ± 0.75***	0.8 ± 0.04	2.6 ± 0.26	1.5 ± 0.13	2.3 ± 0.1**	
	Roots	Control	0.3 ± 0.03	1.9 ± 0.13	1 ± 0.1	2.4 ± 0.18	0.2 ± 0.01	0.5 ± 0.1	0.3 ± 0.02	0.6 ± 0.04	
		WD	0.3 ± 0.03	2.4 ± 0.26	1.2 ± 0.12	1.8 ± 0.17*	0.2 ± 0.01	0.6 ± 0.05	0.4 ± 0.06	0.6 ± 0.06	
	Total biomass	Control	3.6 ± 0.33	16.9 ± 0.99	9.3 ± 1.08	21.1 ± 0.87	1.1 ± 0.08	3.7 ± 0.33	2.1 ± 0.15	4.1 ± 0.23	
		WD	3.8 ± 0.28	16.9 ± 1.25	8.4 ± 0.62	14.1 ± 0.9***	1 ± 0.05	3.2 ± 0.31	1.9 ± 0.19	2.9 ± 0.16**	
Gas exchanges	A	Control	15.8 ± 1.28	8.6 ± 1.22	15.5 ± 0.66	9.1 ± 0.97	11.9 ± 1.56	10.7 ± 1.16	6.4 ± 1.05	10.2 ± 0.91	μmol C0_2_ m^−2^ s^−1^
		WD	13.7 ± 1.44	4.6 ± 2.14	4.2 ± 0.81***	1.7 ± 0.14***	7.6 ± 1.37	7.2 ± 0.75*	4.1 ± 0.82	6.8 ± 1.33	
	E	Control	4 ± 0.48	2.4 ± 0.42	4.4 ± 0.48	2.9 ± 0.33	1.9 ± 0.23	1.7 ± 0.24	1 ± 0.15	1.9 ± 0.28	mmol H_2_0 m^−2^ s^−1^
		WD	2.5 ± 0.36	1.6 ± 0.7	0.5 ± 0.08***	0.3 ± 0.02***	1.1 ± 0.21*	1.3 ± 0.21	**0.6 ± 0.13**	* 0.9 ± 0.17**	
	WUE	Control	4 ± 0.22	3.8 ± 0.32	3.9 ± 0.41	3.3 ± 0.35	6.3 ± 0.26	6.4 ± 0.22	6.3 ± 0.43	5.7 ± 0.29	μmol mmol^−1^
		WD	5.6 ± 0.29**	2.9 ± 0.63	7.4 ± 0.64**	6.4 ± 0.48***	6.7 ± 0.51	5.8 ± 0.34	7.7 ± 0.5	7.1 ± 0.49*	
	g_s_	Control	0.34 ± 0.14	0.17 ± 0.07	0.38 ± 0.12	0.26 ± 0.08	0.13 ± 0.05	0.12 ± 0.04	0.07 ± 0.02	0.13 ± 0.05	mol H_2_0 m^−2^ s^−1^
		WD	0.2 ± 0.08	0.12 ± 0.04	0.04 ± 0.01***	0.02 ± 0.01***	0.08 ± 0.03	0.08 ± 0.03	0.04 ± 0.01	0.06 ± 0.02**	
	δ^13^C	Control	−32.13 ± 2.24	−32.12 ± 1.34	−32.28 ± 2.24	−32.28 ± 2.24	−33.13 ± 1.79	−33.57 ± 2.24	−33.48 ± 1.79	−33.48 ± 1.79	‰
		WD	−31.86 ± 2.24	−31.93 ± 2.24	−30.38 ± 2.24**	−30.04 ± 1.79***	−32.91 ± 2.24	−32.41 ± 2.24***	**−31.34 ± 2.24**	*** ‐30.57 ± 2.24***	
Phytohormones	ABA	Control	38.1 ± 2.9	29.8 ± 3.9	33.7 ± 4.5	29.9 ± 2.1	6.8 ± 0.2	7.1 ± 0.6	7.1 ± 0.3	7.1 ± 0.4	pg mg^−1^ FW
		WD	69.1 ± 6.7**	76.7 ± 10*	148 ± 24**	189.7 ± 18.5**	10.9 ± 0.1*	10.8 ± 0.3**	**16.7 ± 1**	** 38.1 ± 2.3**	
	SA	Control	83.7 ± 16.2	333.5 ± 59	29 ± 4.4	229.5 ± 54.4	78.4 ± 5.8	73.8 ± 3.6	79.3 ± 5.6	64.7 ± 1.8	
		WD	98.2 ± 28.1	33.8 ± 7**	145 ± 74.1	25.8 ± 1.5**	89 ± 4.9	79.1 ± 4.2	103.2 ± 10.7	114.7 ± 11.1**	
Imaging analysis	Side area	Control	336 ± 22	1095 ± 30	872 ± 36	1141 ± 38	236 ± 7	599 ± 12	419 ± 7	630 ± 7	cm^2^
		WD	334 ± 26	1021 ± 38	525 ± 24***	628 ± 27***	233 ± 11	469 ± 13***	**300 ± 7**	*** 330 ± 11***	
		Control	561 ± 27	1427 ± 56	1192 ± 37	1492 ± 90	981 ± 54	2032 ± 66	1492 ± 59	2116 ± 54	
	Convex hull area	WD	576 ± 33	1171 ± 92*	821 ± 42**	1088 ± 53**	1035 ± 47	1688 ± 95**	**1098 ± 63**	*** 1206 ± 47***	
	E × G	Control	0.2 ± 0.004	0.2 ± 0.003	0.21 ± 0.003	0.19 ± 0.002	0.23 ± 0.002	0.25 ± 0.002	0.25 ± 0.002	0.25 ± 0.003	/
		WD	0.19 ± 0.005***	0.18 ± 0.003***	0.15 ± 0.001***	0.15 ± 0.002***	0.23 ± 0.004	0.23 ± 0.002***	**0.22 ± 0.003**	*** 0.21 ± 0.003***	

*Note*: Significant differences in the mean between WD treatment and corresponding control are indicated in bold and using asterisks as follows: *p < .05; ** *p* < .01; ***p < .001. Gas exchange variables and phytohormone concentrations were measured from leaves whose growth occurred during the WD treatment (YLBs). YLBs, young leaf blades; OLBs, old leaves blades; A, assimilation rate of CO_2_; E, transpiration rate; WUE, photosynthetic water use efficiency; g_s_, stomatal conductance; δ^13^C, stable isotope ^13^C abundance; ABA, abscisic acid; SA, salicylic acid; E × G, excess green index.

Table 1 also reports the effects of water deficit on *B. napus* and *T. aestivum* biomass, gas exchange variables, ABA and SA concentrations, as well as on parameters computed from image analysis. Overall, water deficit mainly induced a significant decrease in all cited parameters relative to control plants, except water use efficiency (WUE), ^13^C discrimination (δ^13^C) and ABA concentration, which were increased. At t1_40_, 5 days after water supply was stopped, only the ABA concentration was increased in WD plants of both species (by 1.8 and 1.6 fold in rapeseed and wheat, respectively). The WUE was also slightly increased (1.4 fold) and excess green index (E × G) decreased in rapeseed, while the transpiration rate (E) was significantly reduced in wheat. From a kinetic point of view, at t1_25_ and then t2_25_, the number of significantly affected parameters was progressively increased. Indeed, gas exchange variables (E and δ^13^C), ABA concentration and parameters from images analysis (side area, convex hull and E × G) were strongly affected in both species (as well as assimilation rate (A), WUE and stomatal conductance (g_s_) in rapeseed) as early as t1_25_ (Table 1). Contrariwise, in both species, plants that were maintained at 40% of FC (Figure 1a) showed fewer differences than plants subjected to soil WD reduced to 25% of FC (t1_25_) and then maintained at this level for 9 days (t2_25_). Indeed, only the ABA and SA concentrations associated with convex hull and E × G distinguished WD rapeseed from control plants at t2_40_, while A, δ^13^C and side area also differed significantly in wheat. Since ABA concentration and E × G allowed discrimination of WD from well‐watered pots at each harvest, these parameters seemed to be highly sensitive only 5 days after the restriction of water supply.

### 3.2 Variation in relative net uptake of mineral nutrients induced by water deficit and their concentration in plant tissues

**FIGURE 2:**
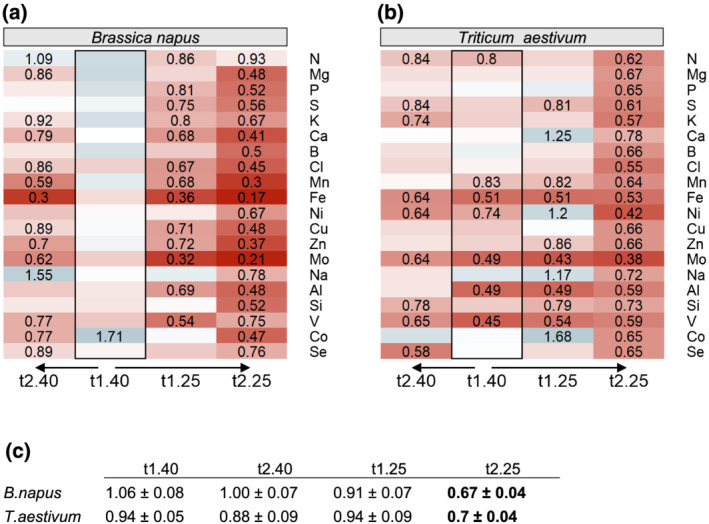
Heatmaps of relative net uptake (NU) from t_0_ by (a) *Brassica napus* and (b) *Triticum aestivum* plants exposed to a soil water content of 40% or 25% of FC during a short (t1) or extended period (t2) compared with control plants maintained at ≥80% of FC. Only significant values for *p* < .05 are given, with a color gradient indicating NU relative to control plants (blue, higher; red, lower). (c) The plant biomass ratios (WD/control) of the total plant dry weight are also given as the mean ± SE (*n* = 5) in order to evaluate the magnitude of NU variations regardless of biomass. Only bold values differed significantly from control plants (*p* < .05). Nutrient net uptake of control plants is provided in Data S4

**FIGURE 3:**
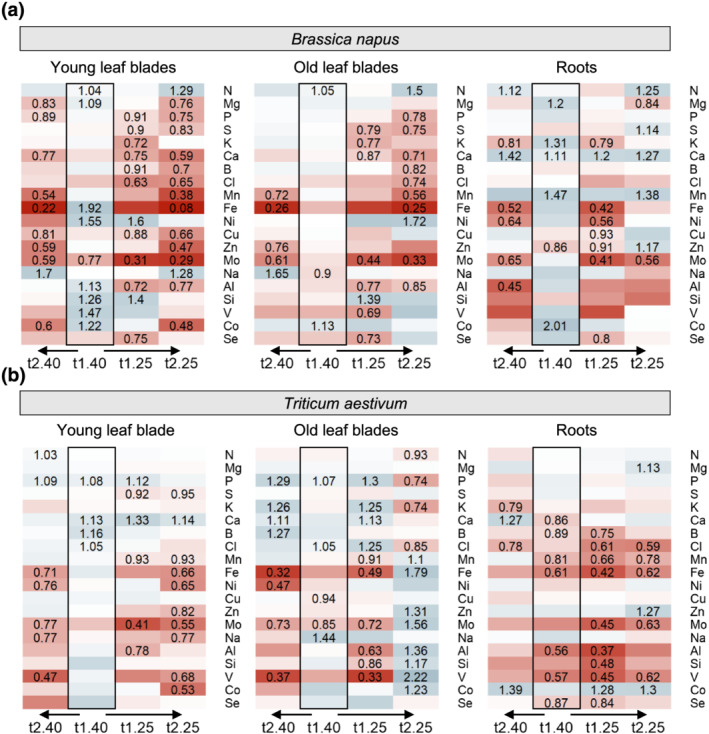
Heatmaps of relative nutrient concentration in (a) *Brassica napus* and (b) *Triticum aestivum* plants exposed to a soil water content of 40% or 25% of FC during a short (t1) or extended period (t2) compared with control plants maintained at ≥80% FC. Only significant values for *p* < .05 are given, with a color gradient indicating relative concentration higher (blue) or lower (red) than control plants. Blank cells in heatmaps correspond to non‐significant variations in concentration compared with control plants. Leaves developed before or after t_0_ are indicated as old leaf blades (OLBs) and young leaf blades (YLBs), respectively. Nutrient concentrations of wheat and oilseed rape tissues (control plants) are given in Data S5

Irrespective of the WD level and duration, water deficit treatments of *B. napus* and *T. aestivum* induced numerous decreases in the relative nutrient Net Uptake (NU) (Data S4) from t_0_ (Figure 2). Nevertheless, at the earliest harvest t1_40_ (5 days after the water supply was stopped), the relative NU in rapeseed was not significantly impaired (except for Co) (Figure 2a). Despite this lack of relative NU variation at t1_40_ (Figure 2a), plant tissue composition (Data S5) was already affected, particularly in YLBs where the Mo concentration was decreased and Fe, Ni, Al, Si, V, and Co concentrations were increased compared with control plants (Figure 3a). Contrastingly, in wheat the relative NU of N, Mn, Fe, Ni, Mo, Al, and V was already significantly affected at t1_40_, and in some cases was reduced by nearly 50% compared with control plants (Figure 2b). This reduction in relative NU led to an ionome disruption that chiefly occurred in wheat roots, where the contents of four out of seven of these elements (Mn, Fe, Al, and V) were significantly reduced (Figure 3b). Nevertheless, except for a slight reduction in Mo content in OLBs, the elemental contents in aboveground tissues were not negatively affected.

Overall, WD induced relative NU reductions in most nutrients, which occurred gradually from t1_40_ to t2_25_ in both species. Among them, Fe, Mo, V, Al, Mn or Zn content showed similar trends in both species (Figure 3). Compared with the 30% total biomass reduction at t2_25_ (WD/control ratio around 0.7) in both species (Table 1), the reduction in NU of most nutrients was much higher in rapeseed; specifically, the uptake of Fe, Mo, Mn, and Zn were reduced by 83%, 79%, 70% and 63% at t2_25_ respectively, with the NU of Fe and Mo having already been affected to the same degree from t1_25_. On the other hand, in wheat the reduction in NU values was similar to biomass for most nutrients, except for Ni, Fe and Mo, which were reduced by a greater amount (Figure 2b). This NU reduction observed at 25% of FC led to massive decreases in the content of a large number of elements in all plant tissues in both plant species, with the exception of wheat OLBs (Figure 3). The most massive effects were found for Fe, Mo, Mn and Zn contents, and mostly in rapeseed. By contrast, plants that were maintained at 40% of FC until t2_40_ showed a lower number of significantly affected NU or nutrient contents, and the ones that were affected were closer to the values observed in control plants than in the t2_25_ WD‐treated plants.

In both species, the relative NU of a few nutrients was transiently increased by water supply restriction. This was the case for Co, Na, Ni, and Ca uptake at t1_25_ in wheat, whereas Co increased in rapeseed at t1_40_ alone (Figure 2). This increased uptake of Co led at the same time, to an increase in root Co content in both species as well as in leaves of rapeseed (Figure 3). A similar trend was also observed for Na in rapeseed, resulting in increased content (1.7 fold increase) in YLBs and OLBs at t2_40_ (Figure 3a).

Although some relative content differed between YLBs and OLBs, no opposing trends in nutrient content dynamics under WD were reported in leaves appeared before or after WD application, regardless of species. However, roots and leaves showed opposite trends for relative concentrations of Ca, Ni, S, or Mn in rapeseed, and to a lesser extent for B, Cl and Co in wheat.

### 3.3 Expression of transport associated genes and KIGs in relation to ionomic changes under water deficit in *B. napus* leaves

**FIGURE 4:**
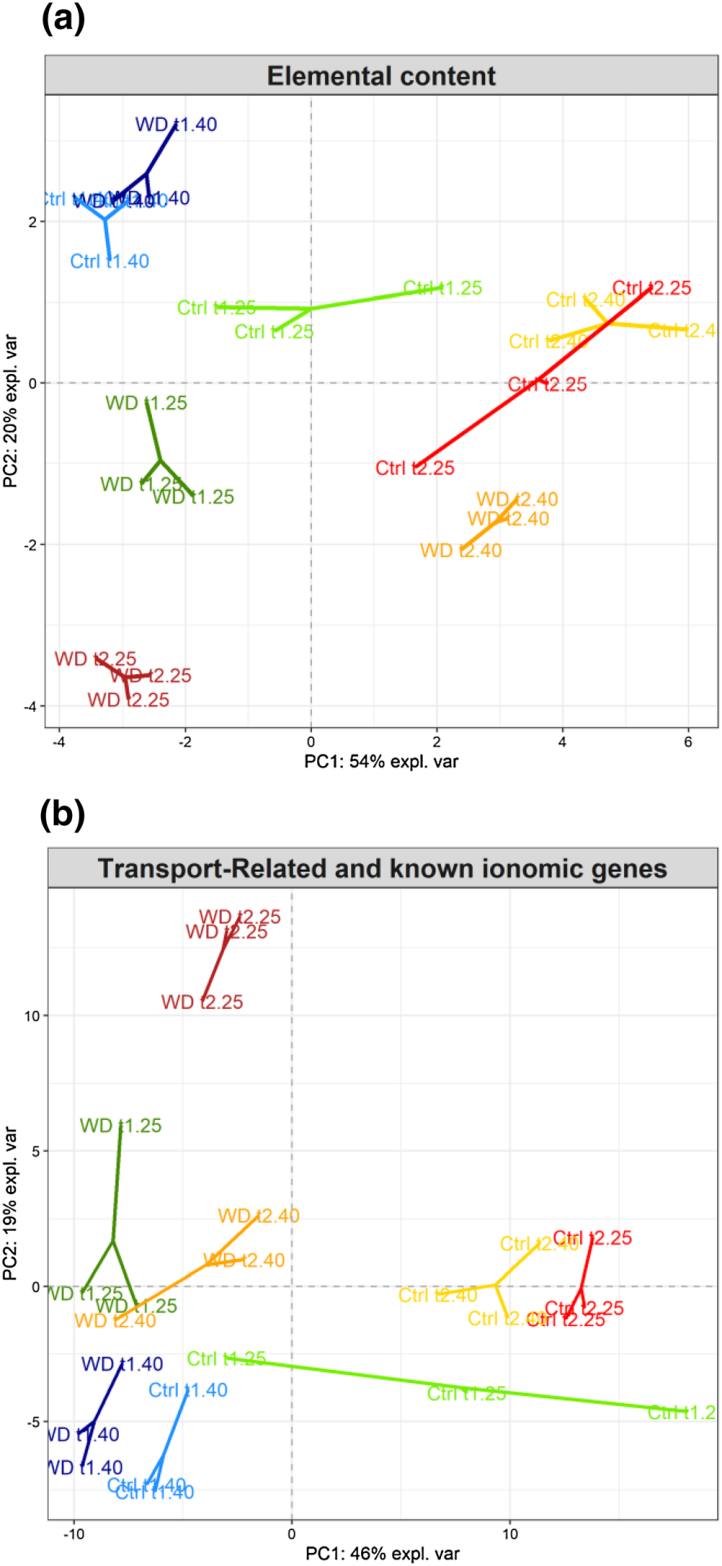
Principal component analysis (PCA) score plots of rapeseed YLBs. (a) Elemental content raw data (ppm) and (b) RNAseq normalized counts of transport‐associated genes (183 genes, among which 103 are known ionomic genes [KIGs]) projected onto the subspace spanned by components 1 (PC 1) and 2 (PC2). Individuals are colored in pairs according to the water regime applied (light, control; dark, WD) at each harvest time

Principal component analysis (PCA) of elemental content (Figure 4a) and the normalized count of transport‐associated genes from RNA sequencing (Figure 4b) in rapeseed YLBs, indicated a high level of profile similarities. Along the first two principal components (PCs), the cumulative proportion of explained variance reached 74% and 66%, respectively, reflecting a similar discriminating power for both types of data. In both PCAs, the first PCs were mainly driven by elapsed time during the experiment and the water deficit applied. WD samples were well discriminated from the respective controls at each harvest, except at t1_40_ where control and WD samples were much closer than the other control‐treatment pairs (Figure 4). In both PCAs, the variability of the control appeared predominantly on PC1 where samples were well segregated from t1_40_ to t2_25_ (Figure 4). In the latter treatment, samples were closer to t2_40_ in accordance with the sampling method whereby only 2 days separated the harvests (Figure 1a). In the same vein, WD samples were well segregated from each other along PC2 in relationship to the level and duration of WD treatments, and besides, both PCAs showed less distance between t1_25_ and t2_40_ compared with other samples. On PC2, elements that contributed the most to sample discrimination (Figure 4a) were P, Co, Se, and Na, with lower P, Co and Se as well as higher Na levels in samples from the lower part of the PCA (loadings available in Data S6) in accordance with previous observations (Figure 3a).

**FIGURE 5:**
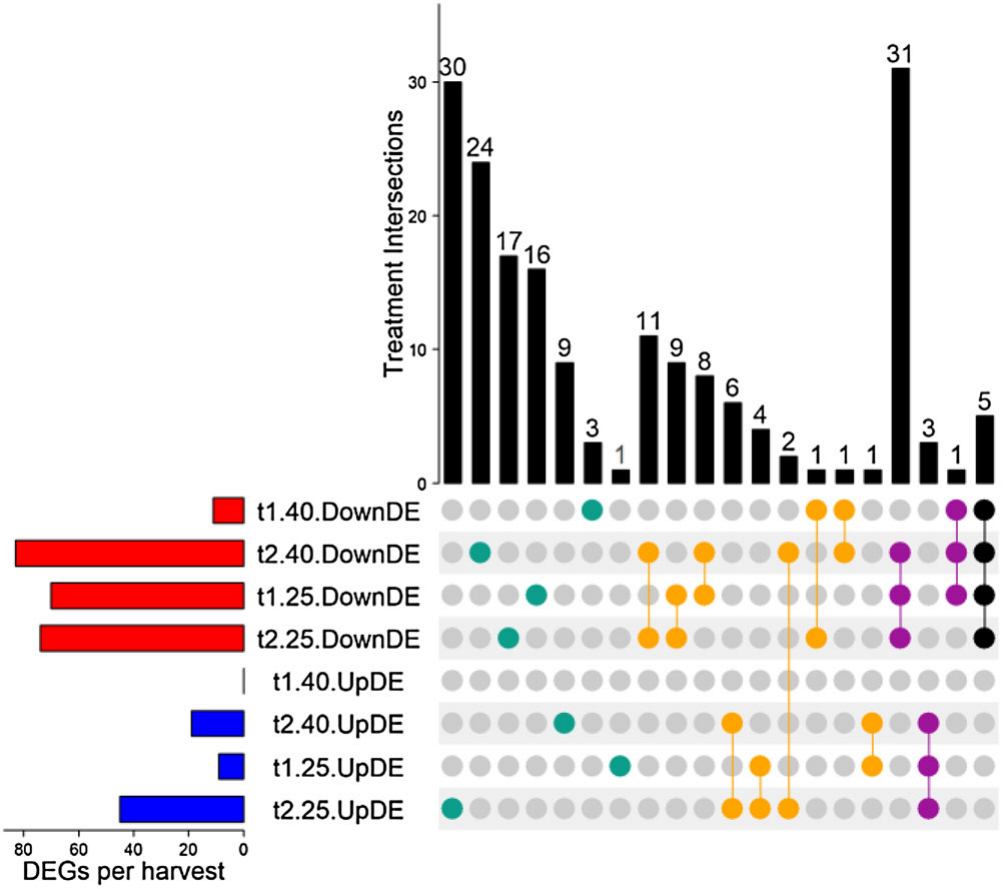
UpSet plot of interactions between upregulated and downregulated differentially expressed transport‐associated genes found in *Brassica napus* exposed to a soil water content of 40% or 25% of FC during a short (t1) or extended period (t2), compared with respective control plants maintained at ≥80% of FC

Among the 183 differentially expressed genes (DEGs) associated with transport functions or identified as KIGs in rapeseed, downregulated DEGs were predominant compared with upregulated DEGs for each harvest (Figure 5). The larger intersection profile corresponded to the expression of 31 DEGs that were specifically downregulated at t1_25_, t2_40_ and t2_25_ (Figure 5). Among them, 43% were related to the transport of a broad group of nutrients comprising Fe, Zn, Cu, and Mn, and a third to P transport, whereas the remaining were linked to N, K, Ca, and Ni transport (Data S3). In addition, 24, 17, and 16 genes related to the same nutrient transports (i.e., P, K, Ca, Fe, Zn, or Mn) appeared specifically downregulated at t2_40,_ t2_25_ and t1_25_, respectively. On the other hand, among genes that were upregulated, around half were found to be differentially expressed at t2_25_. Moreover, 30 DEGs specifically upregulated at t2_25_ (Figure 5) were mainly related to macronutrient transport (59%) and included S, Ca, K, P and Mg (16%, 16%, 13%, 8%, and 6% respectively) as well as Fe, Cu, Zn, and Mn, which in combination reached 37% (Data S3).

**FIGURE 6:**
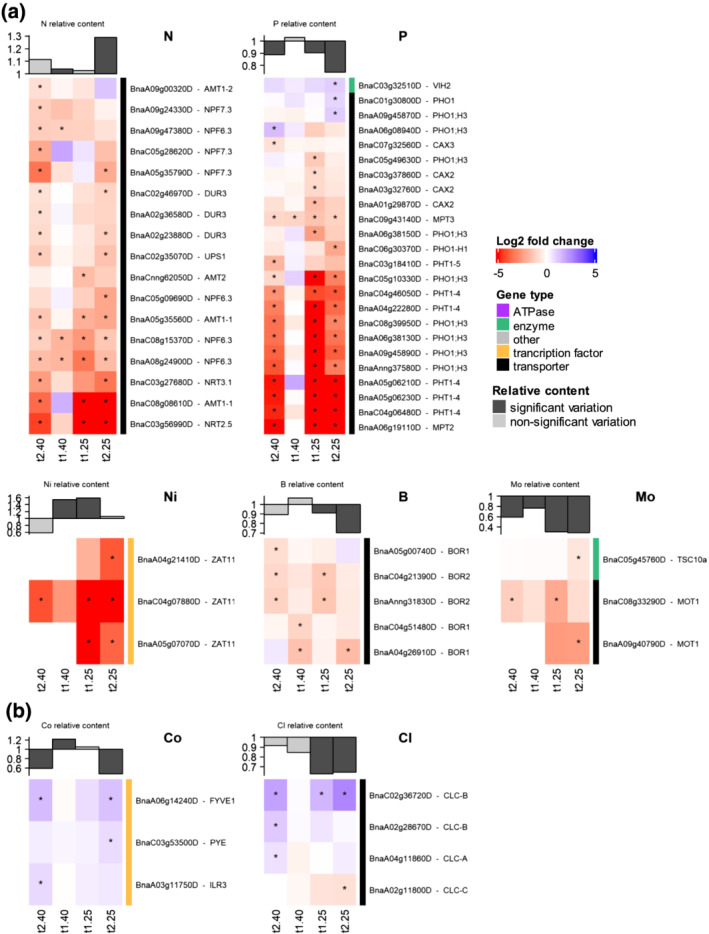
(a) Downregulated and (b) upregulated expression patterns of transport‐related genes and known ionomic genes (KIGs) across WD treatments. Log2 fold changes in differentially expressed genes (DEGs) are indicated with an asterisk “*” for adjusted *p* value <.05. The graph at the top of each panel represents the variation in the relative content (dark gray, significant; light gray, nonsignificant) of the element associated with each set of genes

Interestingly, although these proportions rely on the number of genes tagged for each element, there are contrasting trends when focusing on transporter genes and KIGs related to each nutrient (Figure 6). Several patterns of differential gene expression can be found depending on the nutrient concerned.

**FIGURE 7:**
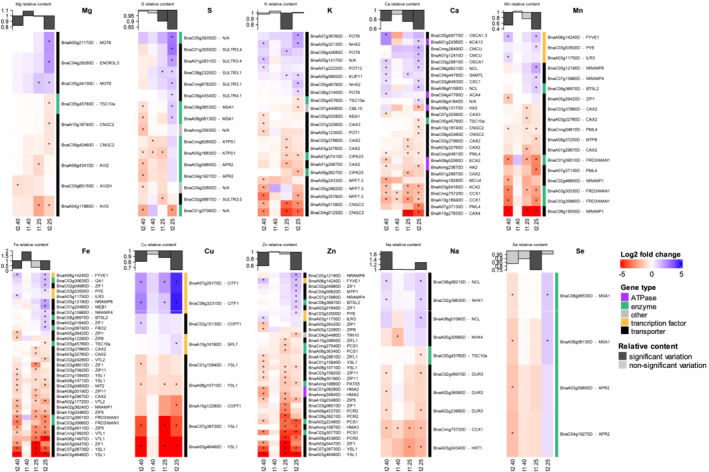
Expression pattern of transport‐related genes and known ionomic genes simultaneously upregulated and downregulated across WD treatments. Log2 fold changes in differentially expressed genes (DEGs) are indicated with an asterisk “*” for adjusted *p* value <.05. The graph at the top of each panel represents the variation in the relative content (dark gray, significant; light gray, non‐significant) of the element associated with each set of genes

The first pattern concerned Cl‐ and Co‐associated genes (Figure 6b), which were mostly upregulated while the Cl and Co contents decreased with the length and intensity of WD. A second different pattern was found for N, P, B, Mo, and Ni transport‐related genes, which were mostly and steadily downregulated (Figure 6a). For example, the transport of all N forms such as ammonium, nitrate or urea was similarly affected, as most of the AMT1, NPF7.3, and NPF6.3 or even DUR3 homolog genes were downregulated, irrespective of the length or intensity of WD (Figure 6a). In the meantime, nitrogen content in the YLBs was higher in WD plants than in controls. For P, this was especially the case for most PHO1‐H3 homologs, as well as PHT1‐4 and MPT2, with some of the largest log2 fold changes relative to control plants (Figure 6a). Lastly, even though the relative Mo content was strongly decreased after t1_40_ in rapeseed YLBs, the MOT1 transporter gene was always found to be downregulated. The third pattern of gene expression was found for macronutrients such as S, Mg, K, and Ca, micronutrients such as Mn, Fe, Cu, and Zn and beneficial elements such as Na and Se (Figure 7). DEGs showed a contrasting profile with simultaneously upregulated and downregulated genes at each harvest time. In such cases, the majority of significantly upregulated genes appeared exclusively at t2_25._ Except for Na, the relative content of these nutrients was mainly decreased in rapeseed YLBs (Figure 3a).

Among the 183 DEGs, only two genes encoding the nuclear protein MSA1 (More Sulphur Accumulation1) and associated with S and Se transport showed a significantly opposite pattern of expression between the two harvest dates (Figure 7). It is the exception which nevertheless emphasizes specific profile found for S and Se in focused heatmaps (Figure 7), which showed opposite patterns of gene expression between t2_40_ and t2_25_. Indeed, two different sets of genes composed notably of ATPS1, APR2 and SULTR3;5 at t2_40_ and SULTR family members (SULTR4;1 SULTR3;1 SULTR3;4) at t2_25_, were mostly downregulated or upregulated, respectively, while the relative S content was significantly lower at t2_25_ only.

## 4 DISCUSSION

Drought, which is usually considered the main abiotic factor affecting plant growth and yield, has been described extensively in the literature in terms of its physiological consequences (Farooq et al., 2009; Nadeem et al., 2019; Raza et al., 2017; Verslues et al., 2006). However, studies considering mineral nutrition remain scarce (Hu & Schmidhalter, 2005; da da Silva et al., 2011; Waraich et al., 2011) compared with those describing the deleterious effects on plant water balance (Tardieu, 2005), stomatal conductance (Tardieu, 2005; Tardieu et al., 2011), oxidative stress (Hasanuzzaman et al., 2013; Lee et al., 2009; Nakabayashi et al., 2014; Sharma & Dubey, 2005), photosynthesis (Reddy et al., 2004) and hormonal balance (Klingler et al., 2010; Loutfy et al., 2012; Park et al., 2021; Shinozaki & Yamaguchi‐Shinozaki, 2007). Hence, the main objective of this work was to obtain a detailed appraisal of the modulation of rapeseed and wheat mineral nutrition in response to a moderate or a severe water deficiency. This was achieved by analyzing the functional ionome coupled with the expression pattern of genes selected from the ionomic gene list curated by Whitt et al. (2020), which is enriched with genes related to transport of mineral nutrients

### 4.1 Drought significantly and precociously reduced mineral nutrient uptake leading to a specific ionomic composition of plants

The short term drought applied here (i.e., 5 days) with a soil WD that dropped from 80% to 40% of FC had already caused interesting ionomic fluctuations in both species before any alteration in most of the physiological parameters. Indeed, the increase in the ABA concentration was almost the only evidence of early perception (at t1_40_) of a lower water availability, which has been previously reported to be a very early response to drought (Ashraf et al., 2013; Klingler et al., 2010). Even though gas exchange variables tended to be altered, the only significant variation observed in rapeseed was an increase in photosynthetic WUE (Table 1), which is also known to be one of the first responses under mild water deficit (Raza et al., 2017). In addition, a similar net uptake of nutrients (Figure 2a) was observed compared with control plants, except in the case of Co, and this indicated that water flow from root to shoot was not yet impaired, which is in accordance with stomatal conductance and ^13^C discrimination (Farquhar et al., 1982) measured in WD‐treated plants (Table 1). At the ionomic level, root (Mg, K, Ca, Mn, and Co) and YLB (N, Mg, Fe, Ni, Al, Si, V, and Co) tissue had already shown elevated element content, whereas in OLBs the elemental contents remained stable (Figure 3). Meanwhile, at the molecular level, very few (11) genes were differentially expressed in YLBs compared with control (Figure 5). These included genes related to N (NPF6.3), P (MPT3), S (ATPS1), Ca (CCX1), B (BOR1), and Na (NHX4) (Figures 6 and 7) and all of them were downregulated.

For the early and moderate water deficit (i.e., t1_40_; Table 1), observations where different for wheat. In fact, probably due to the significant increase in ABA concentration, stomatal conductance (g_s_) and transpiration rate (E) were already significantly decreased, which may lead to a fall in the net uptake of Fe, Mo, Al, and V (decreased by half compared with control), as well as N, Mn, and Ni to a lesser extent. Since wheat is considered to be an efficient species for nutrient remobilization (Maillard et al., 2015), it is possible that the ion partitioning and remobilization during drought that was reported by Etienne et al. (2018) occurred in order to sustain YLBs growth, resulting in decreased root nutrient content (Ca, B, Mn, Fe, Al, V, and Se). In contrast, the P, Ca, B, and Cl content remained stable or increased in YLBs. Under this short‐term and moderate water deficiency (t1_40_), net nutrient uptakes in the two species differed (Figure 2) but resulted in an overall stable content in YLBs, although some contents were increased (Figure 3). Indeed, to ensure development of growing tissue, the leaf ionome is finely tuned and this has been supported by different studies. For example, while growth media was deprived in Fe, its content was not impaired in *Arabidopsis* shoots (Baxter et al., 2008); and in *T. aestivum*, concentrations of transition metal such as Fe, Mn, Cu, and Zn were increased in the early phase of drought (Price & Hendry, 1991).

When soil WD dropped from 80% to 25% of FC (i.e., t1_25_, after 11 days), most of the physiological and morphological parameters (Table 1) shifted towards the typical responses to drought. For example, we observed ABA accumulation and stomatal closure associated with a lower transpiration rate and a decrease in CO_2_ diffusion that led to a reduction in CO_2_ assimilation (Farquhar et al., 1982). Further, a loss of turgor revealed by convex hull analysis as well as disruption of chlorophyll content as measured with the excess green index (E × G) were observed and are widely reported physiological modifications (Tardieu, 2005; Farooq et al., 2009; da da Silva et al., 2011; Simova‐Stoilova et al., 2016; Nadeem et al., 2019), notably in *B. napus* (Raza et al., 2017). These physiological parameters were more deeply impacted after an extended period of WD (i.e., t2_25_) and resulted in a biomass reduction (Table 1) 20 days after the start of water restriction in both species. At the same time, net uptake of nutrients drastically dropped (Figure 2), which was also shown in *S. lycopersicum* (Sánchez‐Rodríguez et al., 2010), leading to a massive decrease in aerial tissue nutrient contents (Figure 3), especially in YLBs. This is in agreement with a previous study reporting a decrease in Ca, Mg, and Fe contents in *A. thaliana* subjected to severe drought (Acosta‐Gamboa et al., 2017). In comparison, plants maintained under an extended mild water deficit (t2_40_) did not show growth cessation (Table 1), but net uptake of nutrients (Figure 2) was also altered (to a lesser extent than t2_25_). As already reported in the literature, these results indicated that plants facing a short and mild water deficit may maintain a transiently stable shoot ionomic content or experience a slight increase in some elements (Acosta‐Gamboa et al., 2017; Fischer et al., 2019) but that a severe water deficit could rapidly affect all nutrients.

Overall, this study clearly demonstrates in two different plant species that mineral nutrition is affected early by water deficit (even a moderate one), before most of the standard parameters used to describe plant responses to drought are affected. More particularly, we found that Fe, Mo, Mn, and Zn content (Figure 2) decreased quickly and sustainably in both rapeseed and wheat, and it is notable that such effects were observed before any significant consequences for growth (i.e., at t1_25_ or t2_40_).

This ionomic fluctuation can be considered as a specific effect of drought on all mineral nutrition, and this is supported by the fact that a substantial number of published articles have reported the drought‐alleviating benefits of remedial nutrient supplies such as foliar application or seed priming (Ghafarian et al., 2013; Kareem et al., 2017; Monjezi et al., 2013; Pourjafar et al., 2016; Zandipour et al., 2018). To the best of our knowledge, these findings originate from empirical approaches under field conditions, where positive effects have been observed on physiological parameters or yield components following compensatory supplies of Fe, Zn (Ashkiani et al., 2020; Monjezi et al., 2013; Zandipour et al., 2018), Mn (Khan et al., 2016), or Mo (Ghafarian et al., 2013; Kareem et al., 2017) in combination or alone, rather than by demonstrations of (i) how drought specifically affects the nutrient uptake and tissue content and (ii) how the nutrient input might counteract it. Indeed, while micronutrients such as zinc (Zn), copper (Cu), manganese (Mn), and iron (Fe) are required in much lower amounts by the plant (Marschner, 2012), they act as metal components or regulatory cofactors in a large number of enzymes involved in photosynthesis and antioxidative metabolism (van van Oijen et al., 2004; Hänsch & Mendel, 2009; da da Silva et al., 2011; Dalcorso et al., 2014; Andresen et al., 2018), which may explain their beneficial effects. For Mo, which is required for enzymes involved in S and N metabolism or in the synthesis of some phytohormones, a positive effect of Mo supply has also been reported in wheat under drought stress, on water uptake via modifications in root morphology and aquaporin expression (Wu et al., 2019).

### 4.2 Patterns of gene expression involved in the ionomic composition of rapeseed were increasingly modified by duration and intensity of drought

In this study, the need to combine ionomic and gene expression analysis revealed some complexities in the results that require cautious interpretation. First, there are differences in the time scales associated with each type of data: (i) Ionomic content is the result of longer term processes (root uptake and transport, accumulation, feedback regulation, modulation by biomass synthesis) and as such can be considered as an integrative set of data, whereas (ii) gene expression provides an instantaneous assessment of current regulation. Second, the interpretation of selected gene expression can exhibit very complex patterns that result from diverse forms of regulation according to each gene's function (root uptake, transport, assimilation enzymes, transcription factors or regulatory genes, among others), while the type of transport (influx or efflux through a given membrane) or the gene's localization (tissue, cellular, and subcellular) may play crucial roles in the interpretation of the overall pattern. Third, gene expression was quantified from leaf tissue and not from roots, and this was partly due to the poor accessibility of the root compartment of plants grown in soil and the difficulty in extracting quality RNA. Nevertheless, the expression of target genes in leaves may be used as a relevant proxy for nutritional status because we found for most nutrients that the root uptake was highly correlated with the leaf nutrient concentration (data not shown) and leaves have been reported as being relevant for determining plant nutritional status (Baxter et al., 2008; DOria et al., 2021).

According to Whitt et al. (2020), the KIG list was limited by the knowledge available at the time and over‐represented with Fe‐ and Zn‐related genes coding transporters and genes that alter the accumulation of these elements. Therefore, in order to decipher potential links between the ionome and the expression of transport‐related genes and KIGs, we hypothesized that the response triggered by upregulation or downregulation of the expression of a given gene could influence the element content in a similar way to an *Arabidopsis* KO or overexpressor mutant, respectively, as noted by Sasaki et al. (2016) and Whitt et al. (2020).

In rapeseed, three expression patterns of transport associated genes and KIGs related to a given element can be observed: (i) Mostly upregulated, such as Cl‐ and Co‐associated genes; (ii) mostly decreased, such as N, P, B, Mo, and Ni transport‐related genes (Figure 6); or (iii) showing a simultaneous upregulation and downregulation, such as genes associated with Mg, S, K, Mn, Fe, Cu, or Zn (Figure 7). For the two latter patterns, while the relative content of these elements was decreased in rapeseed, this was not counterbalanced by upregulation of these transport‐related genes as it could be observed under mineral deficiency. Indeed, he majority of significantly upregulated gene expression appeared exclusively under severe and extended water deficit (i.e., t2_25_), but this group was a minority compared with the overall number of downregulated genes, except in the case of S‐ and Se‐related genes. For Zn, Fe, and Mn, the relative content in YLBs had not yet significantly decreased at t1_25_, which might be related to the late upregulation found later on (t2_25_). However, this was not the case for macronutrient associated‐genes such as S, P, K, or Ca, where the relative content had already decreased at t1_25_, without evidence of upregulation of associated genes. For these elements, the relative content observed might be a result of a complex balance between upregulation and downregulation during the applied WD treatments.

### 4.3 Reduced nutrient uptake and associated modifications of ionomic composition can be explained by changes of gene expression involved in transport

Based on the ionomic composition, which revealed large decreases in most element contents and the modulation of gene expression during increased drought duration or intensity, it is tempting to interpret this with the current knowledge of transport gene regulation under specific nutrient deficiencies. It is generally accepted that the expression of genes encoding nutrient transporters, assimilation enzymes, and transcription or regulation factors, is usually upregulated following nutrient deprivation, or conversely, down‐regulated under nutrient sufficiency. For example, this is the case for N (Krapp et al., 2014), S (*Sultr1.1; 1.2*; Courbet et al., 2019; Shinmachi et al., 2010), K (Ashley et al., 2006; Wang et al., 2013), or Fe (*Irt1*, Vert et al., 2002). In our drought conditions, we found that S uptake (Figure 2) and S tissue content (Figure 3) were strongly decreased, and this was associated with an increased expression of MSA1 (Figure 7), a nuclear localized protein that regulates S homeostasis (Huang et al., 2016) and is associated with an increase in expression of genes encoding SO_4_^2−^ transporters (*Sultr3.1, 3.4, 4.1*). In contrast, N content in the leaves remained stable (or increased) in water restricted plants (Figure 3a), probably as a consequence of increased remobilization of N from old to young leaves, which does not require the induction of the expression of genes encoding N transporters (*AMT1.1, AMT1.2, AMT2*, *DUR3, NRT2.5; NRT3.1, NFP6.3, NFP7.3*) in young leaves (Figure 6a). The case of Fe is more intriguing because its uptake was strongly reduced following drought (Figure 2), resulting in a massive decrease in its content in all tissues (except in young leaves at t1_40_; Figure 3a). According to the literature (Eide et al., 1996; Henriques et al., 2002; Nishida et al., 2012), it is expected that this strong decrease in Fe content should lead to an increased expression of IRT1. However, targeted IRT1 RT‐Q PCR analysis (data not shown) performed on rapeseed shoots revealed no significant difference in IRT1 expression between water‐stressed and control plants. This indicates that Fe acquisition was not controlled through *IRT1* expression under drought as assumed under Fe deficiency (it strongly increased in roots and in leaves), and suggests that other specific mechanisms could be involved during drought. On the ionomic side, several works have revealed numerous crosstalks between Fe and other nutrients (Billard et al., 2014; Courbet et al., 2019; Forieri et al., 2013; Maillard, Sorin, et al., 2016; Vigani & Briat, 2016) and among these interactions are uptake systems that are able to transport Fe as well as other metals. Indeed, we found that several of these genes were downregulated after short‐ and long‐term exposure to severe WD or extended moderate WD (Figure 7). Within this group of genes we can highlight: (i) NRAMP1 (Cailliatte et al., 2010), which may transport Fe, Zn, and Mn; (ii) YSL1 (Waters et al., 2006), which is known to transport ligands and metal such as Fe, Zn, Mn, and Cu; (iii) ZIF1 (Haydon et al., 2012), which encodes a metal ligand transporter; and (iv) FRD3/MAN1, which has a putative role in Fe homeostasis, and whose KO mutants exhibit Fe accumulation (Rogers & Guerinot, 2002). Consequently, we suggest that Fe uptake is downregulated under drought via reduced gene expression, and that secondary reductions occur in the uptake of Mn, Cu, and Zn. Subsequently, that is, after an extended period of severe drought (t2_25_), the low leaf Fe concentration (Figure 3) is associated with the upregulation of genes related to Fe. This was certainly the case for ZIF1, which when over‐expressed in *Arabidopsis* has been shown to lead to higher shoot Fe content (Haydon et al., 2012), and also PYE, a transcription factor that has been reported to positively regulate *Arabidopsis* growth under Fe deficiency (Long et al., 2010). Finally, another study (Rasheed et al., 2016) supports our observations that Fe transport and metabolism are specifically affected by drought, since *Arabidopsis* grown on soil has demonstrated that genes involved in Fe uptake such as IRT1, IRT3, and FRO2 are downregulated early in roots following water restriction.

At this stage, the specific downregulation of Mo uptake observed during drought in this study is more difficult to explain in terms of the regulation involved, other than it being a consequence of a reduction in MOT1 gene expression.

Overall, the results clearly show that root nutrient uptake is specifically affected in the early stages by drought, and that it principally concerns Mo, Fe, Zn, and Mn. However, the identification of specific and early modes of regulation remains difficult, although the changes in the hormonal balance and oxidative status of the plant and/or the soil may be triggering factors (Lee et al., 2007; Park et al., 2021; Rasheed et al., 2016). While in this study, molecular analysis has only performed with oilseed rape, comparative analysis with wheat will require similar investigations at the molecular level.

## CONFLICT OF INTEREST

The authors declare that there are no conflicts of interest

## AUTHOR CONTRIBUTION

A.D.O., A.O., S.D., P.E., M.A., and S.P. designed the experiments. A.D.O. performed the greenhouse experiments and acquired the data. G.C., A.M., and A.O. provided technical assistance to A.D.O. for sample harvest. B.B. performed the treatment of image analysis. L.J. performed phytohormone quantitative analysis. A.D.O. performed RNA extraction and C.P.L.R. performed RNA sequencing analysis. A.D.O. analyzed all data supervised by J.T. and P.E. for transcriptomic data and by A.O. and S.D. for ionomic data. A.D.O. wrote the article, supervised by S.D. and A.O. which completed the writing with contributions of all the authors. All authors approved the submitted version. A.O. agrees to serve as the author responsible for contact and ensures communication.

## Supporting information


**Data S1.** Physical and chemical soil properties
**Data S2**. Composition of the nutrient solution derived from Hoagland nutrient solution and adapted to ensure the plants' mineral needs during the experiment. Elemental concentrations were kept balanced in solution to provide 40 kg N ha^−1^ to ensure a non‐limiting mineral condition for all the experiments.
**Data S4**. Nutrient net uptake (NU) from t_0_ in *Brassica napu*and *Triticum aestivum* control plants maintained at ≥ 80% of FC. Data are expressed as the mean ± SE (n = 25) in μg plant^−1^.
**Data S5**. Mineral nutrient concentration of *B. napus* and *T. aestivum* control plants maintained at ≥ 80% of FC. Tissues developed before or after water deficit treatment are indicated as follows: young leaf blades (YLBs), old leaf blades (OLBs), young petioles (YPs) and old petioles (OPs). Data are expressed as the mean ± SE (n = 25) in parts per million (ppm).
**Data S6**. Principal component analysis (PCA) and loading contribution plots that depict the importance of each element on component 1 (PC1) and 2 (PC), respectively. The bar length represents the regression coefficient with either a positive or negative sign. Variables are ranked according decreasing importance starting from the bottom.

**Data S3.** **List of** transport‐related and known ionomic genes.

## Data Availability

RNAseq data were submitted to the Gene Expression Omnibus (GEO) international repository: http://www.ncbi.nlm.nih.gov/geo accessed on 23 July 2021; GEO accession: GSE179022.
