# Supplementary material for: Drought specifically downregulates mineral nutrition: Plant ionomic content and associated gene expression
Source: Plant Direct. 2022 Aug 5;6(8):e402. doi: 10.1002/pld3.402 (PMC9356223; doi:10.1002/pld3.402)
Supplement: Supplementary file 1 — Data S1. Physical and chemical soil properties Data S2. Composition of the nutrient solution derived from Hoagland nutrient solution and adapted to ensure the plants' mineral needs during the experiment. Elemental concentrations were kept balanced in solution to provide 40 kg N ha−1 to ensure a non‐limiting mineral condition for all the experiments. Data S4. Nutrient net uptake (NU) from t0 in Brassica napuand Triticum aestivum control plants maintained at ≥ 80% of FC. Data are expressed as the mean ± SE (n = 25) in μg plant−1. Data S5. Mineral nutrient concentration of B. napus and T. aestivum control plants maintained at ≥ 80% of FC. Tissues developed before or after water deficit treatment are indicated as follows: young leaf blades (YLBs), old leaf blades (OLBs), young petioles (YPs) and old petioles (OPs). Data are expressed as the mean ± SE (n = 25) in parts per million (ppm). Data S6. Principal component analysis (PCA) and loading contribution plots that depict the importance of each element on component 1 (PC1) and 2 (PC), respectively. The bar length represents the regression coefficient with either a positive or negative sign. Variables are ranked according decreasing importance starting from the bottom. [file PLD3-6-e402-s002.docx]

Soil properties Methods of anaysis Value

Exchangeable CaO (‰) NFX 31-108 - ICP AES 2.14

Total nitrogen (‰) NF ISO 13 878 - Dumas method 1.22

Exchangeable MgO (‰) NFX 31-108 - ICP AES 0.46

P2O5 (‰) NFX 31-160 - Dyer method 0.18

Exchangeable K_2_O (‰) NFX 31-108 - ICP AES 0.093

SO3 (KH2PO4) (mg/Kg) Extr. KH2PO4 - ICP AES 186

P2O5 (‰) NFX 31-161 - Joret Hébert method 0.076

Exchangeable Na_2_O (‰) NFX 31-108 - ICP AES 0.035

P2O5 (‰) NF ISO 11263 - Olsen method 0.048

Cu EDTA (mg/Kg) NFX 31-120 - ICP AES 1.2

Soluble boron Eau (mg/Kg) NFX 31-122 0.16

Fe EDTA (mg/Kg) NFX 31-120 - ICP AES 49.1

Mn EDTA (mg/Kg) NFX 31-120 - ICP AES 23.8

Zn EDTA (mg/Kg) NFX 31-120 - ICP AES 1.4

Clay (‰) NFX 31-107 - without decarbonatation 31

C/N NF ISO 13 878 - Dumas method 42.9

Organic carbon (‰) NF ISO 14 235 52.3

CEC (me/Kg) NFX 31-130 - Metson method 110

Fine silt (s.déc) (‰) NFX 31-107 - without decarbonatation 24

Coarse silt (s.déc) (‰) NFX 31-107 - without decarbonatation 6.1

Organic matter (‰) NF ISO 14 235 90.4

Water pH NF ISO 10 390 6.2

KCl pH NF ISO 10 390 5.9

Fine sand (‰) NFX 31-107 - without decarbonatation 36

Coarse sand (‰) NFX 31-107 - without decarbonatation 903

**Supplementary Data 1**. Physical and chemical soil properties

Salts Salt concentrations (µM) Nutrients Elemental concentrations in solution (µM) input kg ha-1

| KNO3 | 1000 | N | 3500 | 40 |
| --- | --- | --- | --- | --- |
| Ca(NO3)2 +4H2O | 1250 | Mg | 400 | 8 |
| Si(OH)4 | 900 | P | 200 | 5 |
| HCl | 600 | S | 507 | 13 |
| CaCl2 + 2H2O | 500 | K | 1500 | 48 |
| MgSO4 | 400 | Ca | 2110 | 69 |
| CaCO3 | 360 | B | 10 | 0.09 |
| KH2PO4 | 200 | Cl | 2001 | 58 |
| Na2SiO3 | 200 | Mn | 3 | 0.13 |
| K2SO4 | 100 | Fe | 100 | 5 |
| KCl | 100 | Ni | 0.15 | 0.007 |
| NaFe-EDTA  3H2O | 50 | Cu | 0.7 | 0.04 |
| NaFe-EDDHA | 50 | Zn | 3 | 0.16 |
| H3BO3 | 10 | Mo | 0.056 | 0.004 |
| MnSO4 | 3 | Na | 500 | 9 |
| ZnSO4 | 3 | Al |  |  |
| CuSO4 | 0.7 | Si | 1100 | 25 |
| NiCl2 | 0.15 | V |  |  |
| CoCl2 | 0.1 | Co | 0.1 | 0.005 |
| Na2SeO4 | 0.01 | Se | 0.01 | 0.001 |
| (NH4)6Mo7O24 | 0.008 |  |  |  |


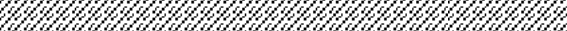

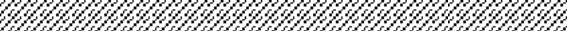
**Supplementary Data 2**. Composition of the nutrient solution derived from Hoagland nutrient solution and adapted to ensure the plants’ mineral needs during the experiment. Elemental concentrations were kept balanced in solution to provide 40 kg N ha-1 to ensure a non-limiting mineral condition for all the experiments.

***Brassica napus Triticum aestivum***

**t1.40**

**t1.25**

**t2.40 t2.25**

489 435 ± 8 875 502 777 ± 7 307

100 253 ± 4 593 116 454 ± 3 955

118 825 ± 3 266 141 920 ± 2 685

224 718 ± 6 471 263 849 ± 3 257

989 043 ± 8 953

522 812 ± 8 627

928 ± 22

547 388 ± 4 555

10 741 ± 565

20 490 ± 1 576

18 ± 0.7

111 ± 2

1 590 ± 78

135 ± 3

103 369 ± 4 580

17 218 ± 457

56 644 ± 3 910

5.9 ± 0.41

18.2 ± 1 4 ± 0.1

**t1.40**

26 363 ± 1 529

1 357 ± 70

5 103 ± 308

1 933 ± 127

37 789 ± 2 220

2 194 ± 113

6.9 ± 0.3

4 202 ± 284

127 ± 7

780 ± 46

1.7 ± 0.1

11 ± 1

37 ± 2

2.6 ± 0.2

387 ± 26

833 ± 68

13 328 ± 844

1.3 ± 0.08

0.66 ± 0.06

0.13 ± 0.01

**t1.25**

63 970 ± 2 612

3 975 ± 154

12 957 ± 530

6 173 ± 297

79 959 ± 3 620

5 644 ± 205

21 ± 1

14 285 ± 689

368 ± 14

1 380 ± 53

3.1 ± 0.1

24 ± 1

100 ± 4

4.8 ± 0.16

978 ± 38

1 782 ± 74

32 235 ± 942

2.4 ± 0.09

1.3 ± 0.1

0.31 ± 0.01

**t2.40**

**t2.25**

N

Mg P

S K

Ca B

Cl Mn Fe Ni Cu Zn Mo Na Al Si V

Co Se

123 689 ± 8 362 372 193 ± 14 807

114 521 ± 4 646 131 992 ± 3 288

9 021 ± 649

15 980 ± 1 005

29 266 ± 2 147

37 259 ± 3 098

58 839 ± 4 103

109 346 ± 6 971

8 572 ± 411

24 242 ± 1 088

10 727 ± 477

9 546 ± 258

29 618 ± 779

11 956 ± 347

147 081 ± 8 720 572 973 ± 28 112 1 069 421 ± 20 025

211 808 ± 8 280 228 772 ± 4 877

53 782 ± 3 587 214 775 ± 12 340

105 ± 8

381 ± 25

27 079 ± 2 411 165 378 ± 17 806

592 ± 47

548 ± 61

2.3 ± 0.2

11 ± 1

115 ± 8

10 ± 1

9 098 ± 593

1 162 ± 118

3 905 ± 452

0.69 ± 0.08

1.4 ± 0.1

0.8 ± 0.06

2 817 ± 290

4 943 ± 651

6.8 ± 0.3

41 ± 3

423 ± 43

60 ± 4

36 839 ± 3 132

6 266 ± 536

17 550 ± 1 426

4.4 ± 0.45

6 ± 0.7

2.3 ± 0.1

466 304 ± 17 614

786 ± 23

441 428 ± 15 930

10 597 ± 450

22 167 ± 1 199

31.2 ± 2.5

93 ± 3

1 429 ± 58

120 ± 4

78 878 ± 2 980

14 007 ± 748

45 223 ± 2 987

6.1 ± 0.4

16.8 ± 0.9

5.1 ± 0.1

13 320 ± 669

42 ± 2

31 629 ± 1 298

832 ± 38

3 112 ± 218

90 ± 2.8

40 ± 2

190 ± 7

26 ± 1.14

1 869 ± 91

3 087 ± 153

64 656 ± 2 919

5.1 ± 0.44

4 ± 0.2

0.69 ± 0.05

14 913 ± 373

53 ± 2

41 843 ± 1 449

931 ± 27

2 377 ± 98

91.6 ± 2.8

44 ± 1

206 ± 6

26 ± 0.7

2 391 ± 111

3 307 ± 157

60 686 ± 1 580

3.7 ± 0.17

4.6 ± 0.1

0.54 ± 0.02

Micronutrients

Macronutrients

**Supplementary Data 4**. Nutrient net uptake (NU) from t_0_ in *B. napus* and *T. aestivum* control plants maintained at ≥ 80 % of FC. Data are expressed as the mean ± SE (n=25) in µg plant-1.

Beneficial nutrients

***Brassica napus***

***t1.40 t1.25 t2.40 t2.25***

|  | **YLBs** | **Yps** | **roots** | **OLBs** | **OPs** | **YLBs** | **Yps** | **roots** | **OLBs** | **OPs** | **YLBs** | **Yps** | **roots** | **OLBs** | **OPs** | **YLBs** | **Yps** | **roots** | **OLBs** | **OPs** |
| --- | --- | --- | --- | --- | --- | --- | --- | --- | --- | --- | --- | --- | --- | --- | --- | --- | --- | --- | --- | --- |
| **N** | 68 504 ± 361 | 50 131 ± 215 | 25 633 ± 254 | 63 069 ± 244 | 49 727 ± 366 | 57 625 ± 915 | 46 386 ± 408 | 20 485 ± 239 | 54 798 ± 1 363 | 45 616 ± 992 | 42 552 ± 572 | 25 647 ± 255 | 15 259 ± 92 | 36 285 ± 826 | 32 994 ± 254 | 35 876 ± 451 | 19 387 ± 235 | 15 321 ± 51 | 29 933 ± 499 | 23 722 ± 417 |
| **Mg** | 3 933 ± 27 | 2 553 ± 17 | 1 094 ± 9 | 4 660 ± 29 | 4 444 ± 51 | 4 746 ± 119 | 3 697 ± 137 | 1 408 ± 33 | 5 532 ± 150 | 4 865 ± 242 | 6 721 ± 135 | 6 168 ± 92 | 1 526 ± 20 | 7 769 ± 231 | 6 604 ± 187 | 6 212 ± 202 | 6 069 ± 203 | 1 645 ± 15 | 7 479 ± 206 | 5 689 ± 163 |
| **P** | 9 431 ± 53 | 6 008 ± 35 | 5 313 ± 34 | 8 237 ± 79 | 4 621 ± 96 | 8 828 ± 97 | 4 953 ± 113 | 5 998 ± 55 | 8 461 ± 198 | 4 606 ± 97 | 8 960 ± 60 | 5 633 ± 26 | 5 982 ± 20 | 9 278 ± 102 | 5 208 ± 57 | 8 421 ± 140 | 5 483 ± 40 | 5 379 ± 98 | 9 487 ± 190 | 4 933 ± 89 |
| **S** | 11 864 ± 98 | 6 454 ± 21 | 5 227 ± 34 | 18 496 ± 123 | 7 481 ± 114 | 14 216 ± 135 | 6 791 ± 128 | 4 569 ± 55 | 21 633 ± 182 | 8 222 ± 278 | 17 742 ± 258 | 6 707 ± 75 | 4 013 ± 31 | 24 257 ± 198 | 8 085 ± 107 | 16 659 ± 364 | 7 046 ± 119 | 4 281 ± 22 | 24 517 ± 444 | 7 784 ± 147 |
| **K** | 48 990 ± 920 | 89 567 ± 1 682 | 24 388 ± 923 | 64 667 ± 937 | 84 953 ± 1 269 | 58 301 ± 1 209 | 96 313 ± 2 166 | 33 216 ± 603 | 79 608 ± 263 | 97 498 ± 2 219 | 38 353 ± 839 | 124 008 ± 1 528 | 28 256 ± 340 | 74 756 ± 1 139 | 112 171 ± 1 817 | 34 161 ± 1 014 | 79 283 ± 1 498 | 24 422 ± 898 | 70 557 ± 1 545 | 62 298 ± 1 394 |
| **Ca** | 18 098 ± 205 | 9 031 ± 58 | 5 049 ± 81 | 31 161 ± 123 | 20 843 ± 246 | 22 993 ± 227 | 12 284 ± 57 | 4 848 ± 71 | 38 624 ± 490 | 21 674 ± 1 033 | 30 734 ± 388 | 20 123 ± 348 | 4 968 ± 48 | 50 392 ± 912 | 25 144 ± 522 | 28 283 ± 657 | 18 361 ± 406 | 5 178 ± 77 | 50 488 ± 1 030 | 22 543 ± 397 |
| **B** | 43 ± 0.3 | 26 ± 0.1 | 18 ± 0.1 | 64 ± 0.7 | 25 ± 0.3 | 50 ± 0.5 | 26 ± 0.1 | 18 ± 0.2 | 74 ± 0.9 | 26 ± 0.3 | 58 ± 1.1 | 28 ± 0.2 | 17 ± 0.1 | 88 ± 1.2 | 25 ± 0.4 | 56 ± 1.3 | 30 ± 0.3 | 17 ± 0.2 | 87 ± 2 | 24 ± 0.1 |
| **Cl** | 7 659 ± 212 | 12 133 ± 374 | 2 298 ± 81 | 13 704 ± 353 | 11 355 ± 280 | 17 026 ± 816 | 21 154 ± 1 249 | 4 323 ± 225 | 22 836 ± 1 019 | 20 674 ± 1 253 | 23 556 ± 438 | 42 056 ± 624 | 5 097 ± 121 | 29 146 ± 601 | 32 162 ± 544 | 23 410 ± 388 | 38 564 ± 1 003 | 5 021 ± 128 | 31 458 ± 411 | 32 128 ± 389 |
| **Mn** | 226 ± 1 | 67 ± 0 | 63 ± 1 | 385 ± 4 | 119 ± 2 | 350 ± 18 | 129 ± 7 | 97 ± 4 | 544 ± 21 | 154 ± 11 | 827 ± 21 | 317 ± 8 | 119 ± 2 | 1 072 ± 32 | 324 ± 7 | 686 ± 41 | 261 ± 13 | 120 ± 3 | 956 ± 41 | 249 ± 13 |
| **Fe** | 155 ± 3 | 131 ± 6 | 678 ± 57 | 180 ± 13 | 318 ± 24 | 409 ± 69 | 83 ± 3 | 2 337 ± 171 | 399 ± 74 | 154 ± 18 | 1 803 ± 68 | 153 ± 5 | 1 402 ± 93 | 2 052 ± 103 | 489 ± 71 | 1 381 ± 121 | 171 ± 6 | 1 072 ± 62 | 1 439 ± 155 | 242 ± 8 |
| **Ni** | 0.61 ± 0.02 | 0.64 ± 0.02 | 2.04 ± 0.13 | 0.55 ± 0.03 | 1.47 ± 0.11 | 0.38 ± 0.01 | 0.92 ± 0.06 | 3.43 ± 0.19 | 0.46 ± 0.01 | 0.8 ± 0.02 | 5.49 ± 0.59 | 0.61 ± 0.04 | 2.56 ± 0.11 | 1.08 ± 0.17 | 1.22 ± 0.16 | 0.82 ± 0.03 | 0.73 ± 0.06 | 2.05 ± 0.09 | 0.63 ± 0 | 0.63 ± 0.03 |
| **Cu** | 7 ± 0.07 | 4 ± 0.05 | 7 ± 0.22 | 6 ± 0.03 | 3 ± 0.06 | 7 ± 0.11 | 3 ± 0.06 | 6 ± 0.05 | 5 ± 0.25 | 3 ± 0.13 | 9 ± 0.16 | 3 ± 0.04 | 6 ± 0.16 | 5 ± 0.06 | 4 ± 0.02 | 7 ± 0.16 | 4 ± 0.06 | 6 ± 0.26 | 4 ± 0.05 | 4 ± 0.09 |
| **Zn** | 76 ± 0.7 | 48 ± 0.4 | 52 ± 0.9 | 49 ± 0.5 | 48 ± 0.3 | 67 ± 4 | 40 ± 1 | 45 ± 0.4 | 45 ± 1.9 | 46 ± 1.7 | 133 ± 3.4 | 50 ± 1.1 | 44 ± 0.3 | 74 ± 2.3 | 71 ± 1.3 | 115 ± 6.4 | 50 ± 1 | 39 ± 0.9 | 66 ± 4.7 | 59 ± 2.4 |
| **Mo** | 4 ± 0.05 | 2 ± 0.03 | 3 ± 0.13 | 5 ± 0.09 | 4 ± 0.05 | 7 ± 0.2 | 4 ± 0.15 | 8 ± 0.4 | 8 ± 0.32 | 6 ± 0.28 | 7 ± 0.19 | 6 ± 0.08 | 5 ± 0.06 | 10 ± 0.3 | 8 ± 0.19 | 7 ± 0.36 | 5 ± 0.16 | 5 ± 0.16 | 10 ± 0.45 | 6 ± 0.16 |
| **Na** | 2 257 ± 35 | 6 063 ± 26 | 1 989 ± 81 | 3 086 ± 47 | 5 872 ± 58 | 3 213 ± 63 | 6 739 ± 145 | 2 622 ± 157 | 3 878 ± 127 | 7 121 ± 220 | 2 959 ± 129 | 8 235 ± 283 | 3 382 ± 73 | 4 532 ± 122 | 8 312 ± 143 | 3 012 ± 98 | 7 455 ± 375 | 4 905 ± 156 | 5 319 ± 194 | 7 846 ± 232 |
| **Al** | 326 ± 2 | 409 ± 5 | 831 ± 37 | 529 ± 17 | 546 ± 26 | 559 ± 12 | 540 ± 23 | 1 797 ± 193 | 696 ± 23 | 613 ± 27 | 636 ± 10 | 921 ± 15 | 1 767 ± 124 | 985 ± 54 | 831 ± 18 | 602 ± 6 | 844 ± 14 | 1 732 ± 116 | 858 ± 10 | 817 ± 14 |
| **Si** | 827 ± 7 | 701 ± 7 | 3 970 ± 328 | 1 554 ± 32 | 1 792 ± 160 | 1 042 ± 26 | 715 ± 8 | 10 029 ± 935 | 1 518 ± 23 | 991 ± 52 | 2 172 ± 217 | 921 ± 17 | 10 609 ± 769 | 4 060 ± 583 | 1 406 ± 78 | 1 242 ± 18 | 824 ± 7 | 13 894 ± 1 303 | 2 148 ± 147 | 1 028 ± 12 |
| **V** | 0.09 ± 0.003 | 0.08 ± 0.003 | 1.2 ± 0.073 | 0.15 ± 0.015 | 0.43 ± 0.037 | 0.05 ± 0.003 | 0.07 ± 0.004 | 4.14 ± 0.535 | 0.13 ± 0.004 | 0.17 ± 0.018 | 0.18 ± 0.021 | 0.08 ± 0.008 | 2.62 ± 0.28 | 0.53 ± 0.096 | 0.24 ± 0.012 | 0.06 ± 0.003 | 0.03 ± 0.001 | 1.8 ± 0.103 | 0.17 ± 0.011 | 0.2 ± 0.009 |
| **Co** | 0.86 ± 0.02 | 0.33 ± 0 | 1.31 ± 0.03 | 0.82 ± 0.01 | 0.34 ± 0 | 0.74 ± 0.06 | 0.29 ± 0.01 | 1.92 ± 0.08 | 0.77 ± 0.03 | 0.28 ± 0.01 | 1.15 ± 0.04 | 0.42 ± 0.01 | 2.4 ± 0.08 | 0.99 ± 0.02 | 0.54 ± 0.02 | 0.99 ± 0.06 | 0.49 ± 0.03 | 2.16 ± 0.07 | 0.81 ± 0.02 | 0.35 ± 0.02 |
| **Se** | 0.28 ± 0.01 | 0.42 ± 0.01 | 0.16 ± 0.01 | 0.38 ± 0.01 | 0.4 ± 0.01 | 0.22 ± 0 | 0.33 ± 0 | 0.25 ± 0 | 0.35 ± 0 | 0.35 ± 0.01 | 0.26 ± 0.01 | 0.48 ± 0.01 | 0.22 ± 0 | 0.3 ± 0.01 | 0.48 ± 0.01 | 0.18 ± 0.01 | 0.22 ± 0 | 0.21 ± 0 | 0.25 ± 0.01 | 0.24 ± 0.01 |

***Triticum aestivum***

**t1.40 t1.25 t2.40 t2.25**

|  | **YLBs** | **roots** | **OLBs** | **YLBs** | **roots** | **OLBs** | **YLBs** | **roots** | **OLBs** | **YLBs** | **roots** | **OLBs** |
| --- | --- | --- | --- | --- | --- | --- | --- | --- | --- | --- | --- | --- |
| **N** | 51 524 ± 177 | 30 242 ± 541 | 48 721 ± 165 | 47 033 ± 226 | 23 507 ± 498 | 41 105 ± 755 | 41 357 ± 173 | 17 679 ± 297 | 37 155 ± 634 | 40 936 ± 163 | 21 089 ± 564 | 36 262 ± 359 |
| **Mg** | 2 109 ± 26 | 1 741 ± 23 | 3 102 ± 27 | 2 383 ± 6 | 1 627 ± 34 | 3 708 ± 37 | 2 611 ± 26 | 1 671 ± 31 | 4 246 ± 51 | 2 599 ± 22 | 1 653 ± 21 | 4 250 ± 35 |
| **P** | 9 368 ± 34 | 6 464 ± 183 | 10 617 ± 90 | 8 861 ± 45 | 5 479 ± 149 | 10 438 ± 95 | 8 213 ± 68 | 4 525 ± 110 | 10 427 ± 100 | 8 289 ± 54 | 5 338 ± 68 | 14 245 ± 70 |
| **S** | 3 566 ± 18 | 2 708 ± 41 | 4 026 ± 22 | 4 187 ± 39 | 2 532 ± 43 | 3 970 ± 34 | 3 605 ± 20 | 2 391 ± 32 | 3 606 ± 28 | 3 481 ± 18 | 2 606 ± 21 | 3 423 ± 21 |
| **K** | 79 569 ± 214 | 35 152 ± 829 | 66 845 ± 394 | 59 759 ± 705 | 31 830 ± 1 083 | 56 857 ± 481 | 77 836 ± 1 472 | 26 504 ± 796 | 40 611 ± 1 004 | 70 410 ± 809 | 32 827 ± 514 | 54 610 ± 1 330 |
| **Ca** | 2 285 ± 35 | 3 784 ± 54 | 4 937 ± 58 | 2 687 ± 17 | 3 552 ± 70 | 6 540 ± 71 | 3 547 ± 43 | 3 934 ± 71 | 7 831 ± 111 | 3 624 ± 29 | 3 356 ± 80 | 8 609 ± 56 |
| **B** | 8 ± 0.14 | 6 ± 0.09 | 16 ± 0 | 11 ± 0.19 | 6 ± 0.07 | 23 ± 1 | 12 ± 0.19 | 6 ± 0.09 | 23 ± 0.23 | 15 ± 0.21 | 5 ± 0.06 | 24 ± 1 |
| **Cl** | 8 843 ± 63 | 3 498 ± 96 | 8 831 ± 56 | 10 173 ± 85 | 3 606 ± 71 | 8 584 ± 45 | 10 811 ± 269 | 4 090 ± 100 | 10 041 ± 156 | 11 950 ± 127 | 5 669 ± 111 | 12 550 ± 202 |
| **Mn** | 144 ± 3 | 217 ± 4 | 256 ± 2 | 179 ± 1 | 242 ± 4 | 356 ± 5 | 225 ± 2 | 260 ± 9 | 420 ± 7 | 224 ± 2 | 276 ± 9 | 390 ± 3 |
| **Fe** | 139 ± 6 | 3 228 ± 184 | 383 ± 21 | 234 ± 16 | 3 176 ± 156 | 482 ± 16 | 477 ± 10 | 2 930 ± 165 | 842 ± 52 | 375 ± 9 | 1 996 ± 89 | 285 ± 14 |
| **Ni** | 1 ± 0.01 | 8 ± 0.3 | 1 ± 0.02 | 1 ± 0.01 | 8 ± 0.3 | 1 ± 0.02 | 31 ± 0.8 | 7 ± 0.2 | 2 ± 0.1 | 28 ± 0.9 | 5 ± 0.1 | 1 ± 0.1 |
| **Cu** | 14 ± 0.1 | 30 ± 1.2 | 15 ± 0.1 | 14 ± 0.1 | 25 ± 0.6 | 13 ± 0.1 | 12 ± 0.1 | 20 ± 0.7 | 11 ± 0 | 12 ± 0.1 | 18 ± 0.3 | 10 ± 0.1 |
| **Zn** | 53 ± 1 | 93 ± 4 | 60 ± 1 | 62 ± 1 | 80 ± 4 | 56 ± 0 | 60 ± 1 | 69 ± 5 | 53 ± 1 | 53 ± 1 | 92 ± 2 | 41 ± 1 |
| **Mo** | 1 ± 0.03 | 8 ± 0.5 | 3 ± 0.04 | 2 ± 0.02 | 7 ± 0.5 | 4 ± 0.03 | 8 ± 0.1 | 6 ± 0.4 | 4 ± 0.1 | 7 ± 0.1 | 5 ± 0.3 | 2 ± 0.02 |
| **Na** | 313 ± 5 | 1 399 ± 78 | 419 ± 11 | 313 ± 3 | 1 689 ± 20 | 656 ± 17 | 314 ± 4 | 1 843 ± 48 | 780 ± 8 | 293 ± 2 | 2 295 ± 126 | 799 ± 25 |
| **Al** | 343 ± 8 | 3 323 ± 178 | 425 ± 13 | 385 ± 10 | 3 966 ± 172 | 672 ± 15 | 456 ± 18 | 4 009 ± 196 | 734 ± 34 | 419 ± 10 | 3 337 ± 204 | 503 ± 12 |
|  |  |  |  |  |  |  |  | 32 844 ± 3 |  |  | 23 218 ± 1 |  |
| **Si** | 9 595 ± 169 | 25 810 ± 1 282 | 28 174 ± 284 | 11 558 ± 190 | 28 556 ± 1 382 | 42 208 ± 469 | 13 267 ± 462 | 053 | 47 460 ± 288 | 12 704 ± 71 | 295 | 41 312 ± 777 |
| **V** | 0.1 ± 0.006 | 6 ± 0.3 | 0.38 ± 0.034 | 0.15 ± 0.012 | 7 ± 0.2 | 1 ± 0.02 | 0.45 ± 0.022 | 7 ± 0.4 | 1 ± 0.07 | 0.28 ± 0.009 | 5 ± 0.2 | 0.4 ± 0.027 |
| **Co** | 0.14 ± 0.003 | 4.22 ± 0.17 | 0.16 ± 0.01 | 0.22 ± 0.003 | 4.12 ± 0.08 | 0.2 ± 0.005 | 0.83 ± 0.02 | 3.81 ± 0.12 | 0.25 ± 0.01 | 0.92 ± 0.02 | 3.21 ± 0.08 | 0.21 ± 0.01 |
| **Se** | 0.18 ± 0.004 | 0.28 ± 0.01 | 0.2 ± 0.003 | 0.21 ± 0.01 | 0.25 ± 0.001 | 0.15 ± 0.003 | 0.21 ± 0.02 | 0.22 ± 0.01 | 0.24 ± 0.01 | 0.13 ± 0.002 | 0.23 ± 0.003 | 0.24 ± 0.002 |

**Supplementary Data 5**. Mineral nutrient concentration of B. napus and T. aestivum control plants held at ≥ 80% field capacity level. Tissues developed before or after water deficit treatment are indicated as follows: young leaf blades (YLBs), old leaf blades (OLBs), young petioles (YPs) and old petioles (OPs).

Data are expressed as the mean ± SE (n=25) in parts per million (ppm).


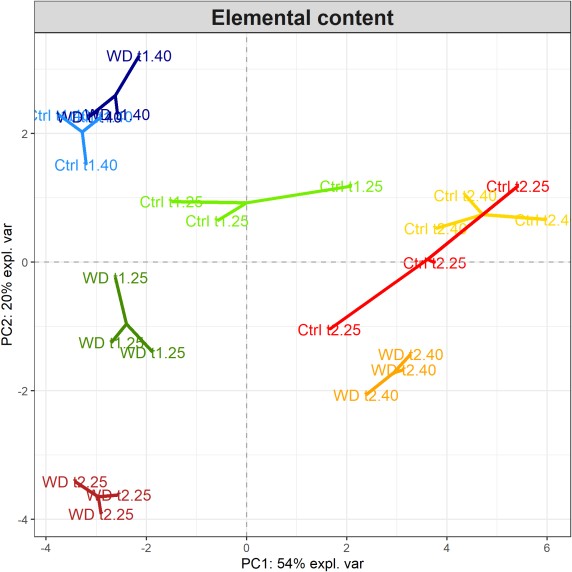


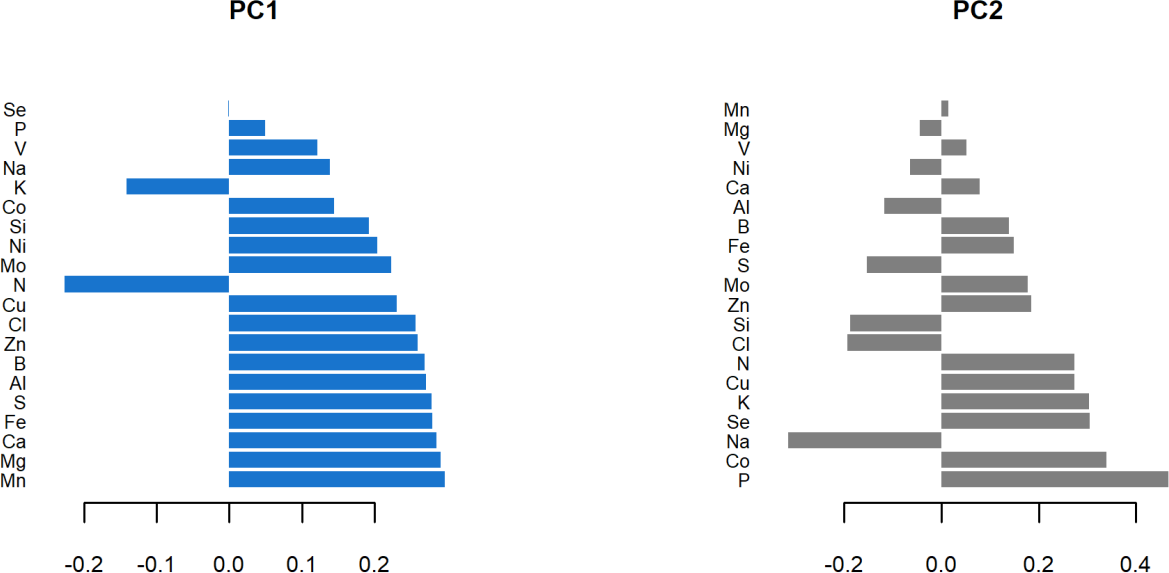


**Supplementary Data 6**. Principal component analysis (PCA) and loading contribution plots that depict the importance of each element on component 1 (PC1) and 2 (PC), respectively. The bar length represents the regression coefficient with either a positive or negative sign. Variables are ranked according decreasing importance starting from the bottom.
